# Atomic-level molybdenum oxide nanorings with full-spectrum absorption and photoresponsive properties

**DOI:** 10.1038/s41467-017-00850-8

**Published:** 2017-11-16

**Authors:** Yong Yang, Yang Yang, Shuangming Chen, Qichen Lu, Li Song, Yen Wei, Xun Wang

**Affiliations:** 1 The Key Laboratory of Organic Optoelectronics and Molecular Engineering, Department of Chemistry, Tsinghua University, Beijing, 100084 China; 2 The Key Laboratory of Bioorganic Phosphorus Chemistry & Chemical Biology (Ministry of Education), Department of Chemistry, Tsinghua University, Beijing, 100084 China; 3 National Synchrotron Radiation Laboratory, Hefei Science Center CAS, University of Science and Technology of China, Anhui, Hefei, 230029 China

## Abstract

Superthin nanostructures, particularly with atomic-level thicknesses, typically display unique optical properties because of their exceptional light–matter interactions. Here, we report a facile strategy for the synthesis of sulfur-doped molybdenum oxide nanorings with an atomic-level size (thickness of 0.5 nm) and a tunable ring-in-ring architecture. These atomic-level nanorings displayed strong photo-absorption in both the visible and infrared-light ranges and acted as a photothermal agent. Under irradiation with an 808 nm laser with an intensity of 1 W/cm^2^, a composite of the nanorings embedded in polydimethylsiloxane showed an ultrafast photothermal effect, delivering a local temperature of up to 400 °C within 20 s, which to the best of our knowledge is the highest temperature by light irradiation reported to date. Meanwhile, the resulting nanorings were also employed as a photoinitiator to remotely induce a visible-light shape memory response, self-healing, reshaping performance and reversible actuation of dynamic three-dimensional structures. This study demonstrates an advancement towards controlling atomic-level-sized nanostructures and achieving greatly enhanced optical performances for optoelectronics.

## Abstract (web summary)

Nanostructures with atomic-level thicknesses have the potential to exhibit unique size-dependent properties. Here the authors fabricate sulfur-doped molybdenum oxide architectures with tunable ring-in-ring morphologies and strong visible- and IR-light absorption, endowing them with a range of photoresponsive properties.

## Introduction

One of the essential targets of nanoscience is to understand the size-dependent properties of materials, which have led to many important research fields exploiting quantum dots^1–3^, surface plasmon resonance^4, 5^, and low-dimensional nanostructures^6–8^. However, with the gradual enrichment of the nanomaterial family, it becomes more and more difficult to expect new size-related phenomena by simply reducing sizes to nanometer scales. As a logical expansion, further processing of materials to their size limit on the atomic level leads to a dramatic increase of the exposed free charges, which can be accompanied by unprecedented properties including photothermal conversion^9, 10^ and optomechanical actuators^11, 12^. Accordingly, new plasmonic behaviors or optoelectric properties can be expected for atomic-level nanomaterials, which thus would be beneficial to explore and extend their optical properties.

Recently intense research attentions has been paid to photo-responsive composites because of their great potentials in a wide range of micro- and nano-sized devices^13, 14^, such as photonic switches^15^ and micro-optomechanical systems^16, 17^. The incorporation of a photothermal conversion material into the polymer is an effective method to prepare a composite with light-driven performances such as shape memory, reconfiguration, or healing^18^. Consequently, the key to this functionality is the presence of an efficient photothermal material, effectively absorbing light and converting photon energy into heat, which increases the local temperature and drives chemical reactions. To date, the well-known photoactuators primarily include organic compounds, e.g., azobenezenes^19^, carbon-based materials including carbon nanotubes and graphene^20, 21^, liquid crystal elastomers^22^ and transition metal dichalcogenides^23^. However, most photoactuators suffer from poor compatibility with polymers^24, 25^ and relatively low photothermal conversion efficiency with narrow absorption ranges. Recently, molybdenum-based nanomaterials have gained significant attentions due to their unique optical properties and intrinsic electronic structures with high carrier mobility^26, 27^, which allow for the conversion of photons into mechanical motion or other energy^28^. Besides transition-metal dichalcogenide, molybdenum dioxide (MoO_2_), as a metallic semiconductor, can be used as an alternative plasmonic material, owing to its intrinsic electronic structure that exhibits unique plasma frequencies^29^. In this case, new plasmonic behaviors or optoelectric properties are expected for molybdenum oxides nanostructure with atomic-level sizes due to their all-surface-atom microstructures.

Herein, we report a class of hierarchical sulfur-doped MoO_2_ nanorings (SMO NRs) with atomic-level thickness and a tuneable ring-in-ring architecture using a robust and large-scalable strategy. Such a ring-in-ring architecture displayed a unique geometrical diversity and self-assembly property. By simply adjusting the doping quantity, the number of nanorings (NRs) was tuned from a multi-level ring (mSMO NRs) to a single ring (sSMO NRs). Taking advantage of the strong light absorption as well as the excellent polymer compatibility, the resulting sSMO NRs acted as photothermal transduction agents in a polymer, possessing strong visible and NIR-light absorbance and efficiently converting light energy into heat. As a result, under irradiation by an 808-nm laser, these NRs embedded in a polydimethylsiloxane (PDMS) composite showed an ultrafast and impressive photothermal effect, generating a local temperature of up to 400 °C within 20 s, which is the best reported heating performance by light irradiation. Localized chemical transesterification was remotely triggered by introducing NRs into an epoxy, resulting in visible-light-activated shape memory within seconds. Meanwhile, self-healing and shape-deformation performances were also achieved within minutes under the remote control of infrared laser light. Notably, such a light-induced method also allowed to manipulate the alignment of liquid crystalline elastomers with exchangeable links (xLCEs), leading to a reversible actuation for dynamic 3D structures. The atomic-level NRs reported here are promising materials for future micro-optomechanical systems and intelligent applications.

## Results

### Structure and composition characterization

**Fig. 1:**
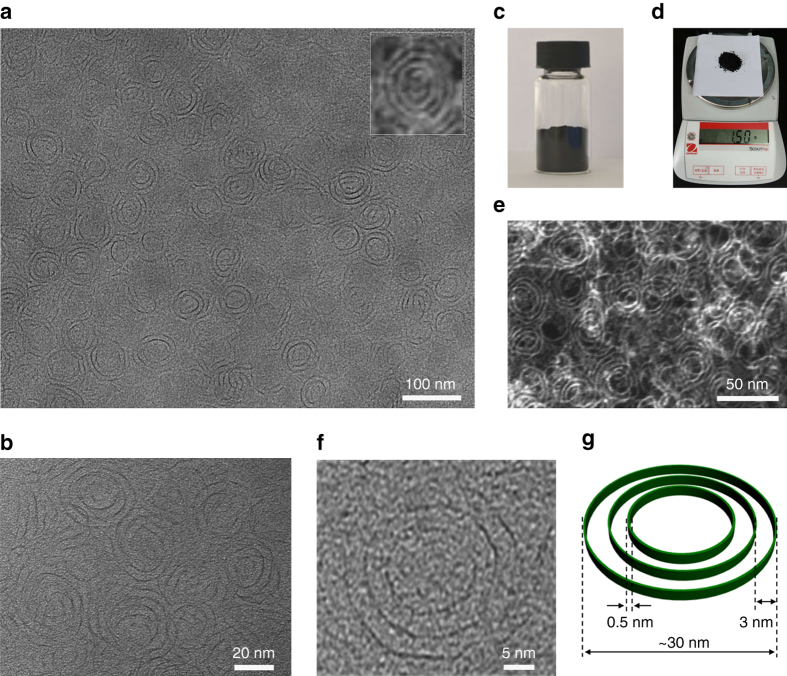
Morphology characterization of the mSMO NRs. **a**, **b** Scanning electron microscopy images of the as-synthesized mSMO NRs with a yield of 100%; the inset shows a typical image of the free-standing mSMO NRs, displaying a unique ring-in-ring shape; **c** photograph of the as-synthesized sample; **d** photograph of the products obtained in a large quantity of 1.5 g in a 100-mL autoclave; **e** HAADF-STEM image of the mSMO NRs; **f** HRTEM image of the mSMO NRs; **g** the corresponding structural model of the mSMO NRs; the thickness of the ring is about 0.5 nm; the outermost NR size is  ~ 30 nm; the interval distance between the NRs is  ~ 3.0 nm

An one-pot solvothermal approach was developed to produce the hierarchical NRs by using ammonium heptamolybdate and thiourea as the precursors and oleylamine and oleyl alcohol as a surfactant and solvent. Briefly, ammonium heptamolybdate and thiourea with controlled molar ratios were dissolved in water and mixed with oleyl alcohol and oleylamine. After reaction, the black products were centrifuged and separated for characterization. The resulting product was initially characterized by transmission electron microscopy (TEM) and high-angle annular dark-field scanning TEM (HAADF-STEM). As shown in Fig. 1a, b, the as-synthesized sample was composed of numerous well-defined NRs. The HAADF-STEM image clearly reveals large areas with perfect, free-standing NRs (Supplementary Fig. 1). Careful observations revealed that the NRs possessed unique ring-in-ring shapes in a flat plane. The ring size gradually decreased from the exterior to interior. The diameter of the exterior NRs was 30 nm, and the interval distance of the NRs was  ~ 3.0 nm (Supplementary Fig. 2). Notably, the NRs were the only product formed with an impressive yield of 100%. Strikingly, this synthetic method is easily scalable, and as a demonstration, a large quantity of sample was yielded on the gram scale (Fig. 1c–e).

The composition of the as-synthesized NRs were characterized using powder X-ray diffraction (PXRD), where the two broad peaks indicated the amorphous nature of the sample (Supplementary Fig. 3a). The Mo to S atomic ratio measured by inductively coupled plasma atomic emission spectroscopy (ICP-AES) analysis was quantitatively determined to be 5:1 (Supplementary Table 1). The structure of nanorings was further studied by High-resolution transmission electron microscopy (HRTEM), as shown in Fig. 1f. Close examination revealed an amorphous structure with a ring-in-ring geometry, which is consistent with the PXRD results. With a tilt angle of  + 20°, the side view of the ring-shape structure clearly depicts the morphology, and the thickness of the NRs was only 0.5 nm (Supplementary Fig. 4). Owing to their atomic-level thickness, the SMO NRs were sensitive to electron beam irradiation, giving rise to electron-beam-induced damage and crystallization (Supplementary Fig. 5). The as-prepared NRs were also characterized by Raman spectroscopy, FTIR spectroscopy and thermal gravimetric analysis (TGA). In Raman spectroscopy (Supplementary Fig. 3c), two characteristic peaks located at 560.5 and 726.3 cm^−1^ were assigned to the telescopic vibration mode of molybdenum dioxide^30^. On the basis of the above results, the as-syntheiszed sample was considered as multi-level sulfur-doped molybdenum oxide NRs (mSMO NRs, Fig. 1g).

**Fig. 2:**
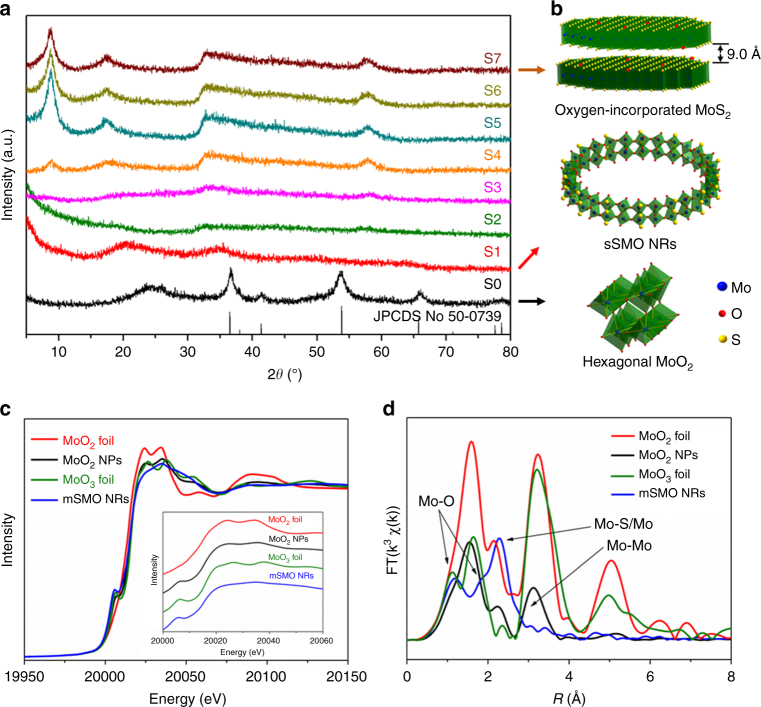
Structure analysis of the mSMO NRs. **a** Evolution of the XRD patterns with various Mo/S molar ratios; **b** the corresponding structural models of the hexagonal MoO_2_ NPs, the SMO NRs and oxygen-incorporated MoS_2_ with enlarged interlayer spacing; **c** XANES patterns and **d** FTs of the Mo K-edge EXAFS patterns of the MoO_2_ NPs, the mSMO NRs, MoO_2_ foil and MoO_3_ foil

The most striking feature of this synthesis was the ring-in-ring nanostructures in a horizontal plane. Until now, almost all NRs have possessed single-loop geometries with rigid structures, and the self-assembly of a multi-level ring structure has never been reported. In the synthetic process, sulfur doping was a key factor for the formation of the unique NRs structure. The sample obtained without adding thiourea was found to contain well-crystallized hexagonal MoO_2_ nanoparticles (MoO_2_ NPs, Supplementary Fig. 6 and 7). Considering the larger atomic radius of sulfur, sulfur will enter the lattice and occupy the position of the oxygen atoms, resulting in a crystalline to amorphous transition. Increasing the thiourea content resulted in a continuous structural evolution from the hexagonal MoO_2_ phase to the oxygen-incorporated MoS_2_ phase^31^. The corresponding evolution of the PXRD patterns with different Mo to S molar ratios is shown in Fig. 2a. With an increase of S to Mo molar ratio, an interesting structural evolution from nanoparticles to NRs and to a lamellar structure can be observed on the basic of TEM images (Fig. 2b and Supplementary Fig. 8). Excess sulfur doping led to an oxygen-incorporated MoS_2_ lamellar structure (Supplementary Fig. 9). Clearly, sulfur doping played an important role in the final structure and morphology of the product.

### X-ray absorption spectroscopy studies

To further identify the structure of the mSMO NRs, X-ray absorption spectroscopy (XAS) was utilized to analyze the specific coordination structure and the atomic environment. Figure 2c, d shows the Mo K-edge X-ray absorption near edge structure (XANES) spectra and the Fourier transform (FT) of the Mo K-edge extended X-ray absorption fine structure (EXAFS) spectra for the as-synthesized mSMO NRs, MoO_2_ NPs, MoO_2_ foil, and MoO_3_ foil. The XANES spectral shape (the pre-edge and white-line peaks) of the mSMO NRs showed obvious broadening compared with that of the MoO_2_ NPs (Fig. 2c), indicating a more disordered structure for the mSMO NRs. The FT of the EXAFS data showed that the coordination in the mSMO NRs was different from those of the MoO_2_ NPs and MoO_2_ foil, reflecting the complex crystal structure of the mSMO NRs (Fig. 2d). Two types of Mo–O bonds, two types of Mo–Mo bands and one type of Mo–S bonds were present after sulfur doping. Mo K-edge EXAFS analysis revealed that sulfur doping generated a Mo–S bond at *R = *2.41 Å and a Mo–Mo bond at *R = *3.01 Å. The detailed structural parameters are summarized in Supplementary Table 2. The EXAFS results provide solid evidence that the prepared mSMO NRs possess similar structural components as MoS_2_. Compared with the MoO_2_ NPs, the introduction of sulfur into the lattice, occupying the positions of the oxygen atoms, results in the formation of Mo–S bonds and the lattice distortion. Accordingly, lattice stress caused by sulfur doping led to the transformation from a crystalline to amorphous ultrathin structure.

### Growth mechanism

The conformations diversity of ultrathin nanostructures highly depends on the energy balance between the organic ligands and the inorganic backbone structures^32–34^. We believed that the atomic-level thickness of the nanostructure is a prerequisite for the formation of the self-cyclization structure. According to the principle of the lowest energy, the formation of the bending structure for flexible one-dimensional materials is favorable if the bending energy (*E*_*b*_) is larger than the rigidity energy (*E*_*r*_). *E*_*b*_ usually involves lattice stress of the structure and ligand interactions, and E_*r*_ is corresponds to the strain energy (*E*_*s*_). The strain energy can be calculated by the following formula: *E*_*s*_ = (*YI*)·*L*/2*R* ^2^, where *Y* is the Young’s modulus, *YI* represents the flexural rigidity, which is dependent on the thickness of the structure, *L* is the length of the bending structure, and *R* is the radius of the resulting bending structure^35^. Clearly, a thinner structure will result in more flexibility. On one hand, reduction of the atomic-level size can dramatically decrease the rigidity of the material and lower the bending energy. On the other hand, lattice stress induced by sulfur doping increases the bending energy to surpass the rigidity energy, leading to self-cyclization. In this case, as the sulfur doping content increases, the bending stress gradually increases, leading to the formation of single-level NRs with a smaller radius owing to the curvature increase. Therefore, the lattice stress generated by ion doping is the key contributor to the number of NRs.

**Fig. 3:**
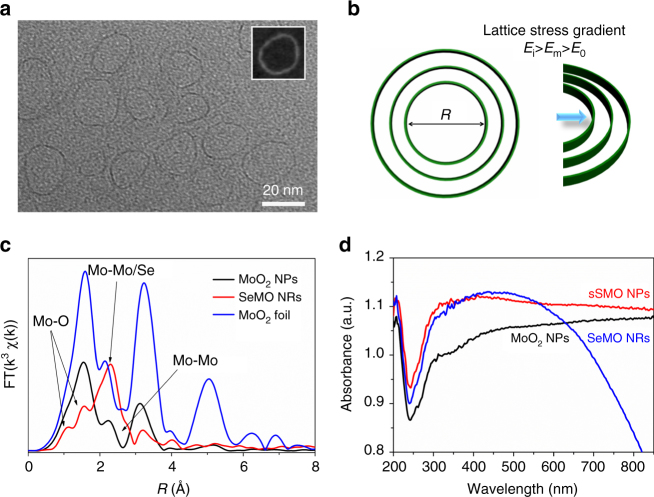
Formation mechanism of the NRs. **a** TEM image of the single sSMO NRs; the inset shows the HAADF-STEM image of the single NRs; **b** lattice stress gradient analysis of the multi-level NRs; *E*_*i*_, *E*_*m*_ and *E*_*o*_ represent the strain energy of the interior NRs, middle NRs and exterior NRs, respectively; **c** FT spectra of the k3-weighted Mo K-edge EXAFS of MoO_2_ foil, the MoO_2_ NPs, and the SeMO NRs; **d** UV–vis diffuse reflectance spectra of the sSMO NRs, MoO_2_ NPs and SeMO NRs

Interestingly, well-defined single NRs (sSMO NRs) can be harvested by fine tuning the amount of sulfur doping. The typical conformation of the sSMO NRs are shown in Fig. 3a, revealing free-standing single NRs with an average diameter of 18 nm. Notably, two similar broad peaks were observed in the PXRD pattern (Supplementary Fig. 10), indicating the amorphous structure of the sSMO NRs. The Mo to S atomic ratio of sSMO NRs was quantitatively determined to be 2.2:1 based on ICP-AES analysis, indicating a higher content of sulfur doping than that in the mSMO NRs. On the basis of the XPS spectrum in Supplementary Fig. 11, more Mo^4+^ states of MoS_2_ were formed in sSMO NRs with an increment increase of sulfur doping, resulting in the greater lattice stress. As discussed above, increased sulfur doping results in higher lattice stress, leading to the formation of single NRs. We intuitively hypothesize that a lattice stress gradient exists, which decreases from the interior to the exterior: *E*_*i*_ > *E*_*m*_ > *E*_*o*_, in the mSMO NRs (Fig. 3b and Supplementary Fig. 12). A larger lattice stress result in NRs with a smaller radius. Accordingly, changing the sulfur doping enables to tune the lattice stress, thus ultimately resulting in a tuneable ring-in-ring hierarchical architecture.

On the basis of the lattice stress effect, we proposed that the NRs structure could also be prepared by selenium doping. As expected, a similar ring structure was successfully synthesized by selenium doping (denoted as SeMo NRs). As shown in Supplementary Fig. 13, the as-obtained products exhibited a clear multi-level ring structure. The detailed characterizations, including PXRD, ICP-AES and XANES, confirmed the formation of an amorphous structure after selenium doping. XAS was also performed to verify the Mo coordination environment after selenium doping. The in-depth EXAFS analysis, shown in Fig. 3c, demonstrated that two types of Mo–Mo bonds and one type of Mo–Se bonds were formed (Supplementary Table 3). Clearly, the Mo–Se bond at *R* = 2.50 Å and the Mo–Mo bond at *R* = 3.02 Å were observed after doping, indicating similar structural features as MoSe_2_.

The growth process of the mSMO NRs was further investigated by time-dependent TEM analysis (Supplementary Fig. 14), indicating that multi-level NRs were obtained in the initial stages of reaction. As time progresses, the ring-in-ring architecture did not change but the yield increases, indicating the simultaneous growth of ultrathin nanostructures during the self-cyclization process. Interestingly, single NRs were also successfully prepared by tuning the solvent ratio. We believed that alkylamine plays a crucial role in the self-cyclization and the hierarchical architecture, not only acting as a capping ligand to form the atomic-level structure (Supplementary Fig. 15) but also serving as a bridge to self-assemble NRs. An increase of the amount of oleylamine results in a larger steric repulsion between the NRs, leading to the formation of single NRs. For the ring-in-ring architecture, van der Waals forces between ligands provide stability for the hierarchical assembled structure. Meanwhile, this non-bonding force between the NRs can be weakened or even destroyed by centrifugation, thus followed by the detachment of the multi-level NRs (Supplementary Fig. 16). Therefore, ligand interactions on the surface of the NRs not only provide a driving force to assemble the NRs but also maintain the stability of the free-standing ring-in-ring architecture. Unlike the coils of carbon nanotubes that are induced by external energy^36^, the driving force for the NR self-cyclization originates from the internal lattice stress caused by sulfur doping. We proposed that the formation of the ring-in-ring nanostructure undergoes two steps including self-cyclization and the subsequent self-assembly process. The spontaneous formation of the mSMO NRs is attributed to the synergetic effect of the lattice stress induced by ion doping and the ligand–ligand interactions.

### Photothermal and light-responsive performance

UV–vis-NIR absorption spectra of the sSMO NRs and mSMO NRs dispersions shows a broad absorption bands spanning the visible and NIR regions (Supplementary Fig. 17a), which is consistent with previous reports on graphene oxide and metallic transition metal dichalcogenides^37, 38^. In addition, the sSMO NRs dispersion showed enhanced light-absorption intensity compared with that of the mSMO NRs dispersion, which was directly related to the electronic structure of the sSMO NRs. On one hand, the amorphous NRs have unique atomic arrangements and distinct electronic properties owing to the short-range order^39^. On the other hand, sSMO NRs possess more free electrons after more sulfur doping, leading to a strong light–matter interaction^40^. The enhanced light-absorption effect of the sSMO NRs is attributed to the synergistic effect of the amorphous and plasmonic structure. Moreover, the difference in absorption properties is more evident from the light-induced photothermal effect because of the notable absorption at  ~ 808 nm in the NIR region. The temperature elevation indicates that the photothermal effect of the sSMO NRs is higher than that of the mSMO NRs (Supplementary Fig. 17b). The UV–vis diffuse reflectance spectra of the samples is further shown in Fig. 3d. The sSMO NRs exhibited higher light absorption as compared with the MoO_2_ NPs and SeMO NRs. The strong optical absorption indicates that the sSMO NRs can be employed as a photoinitiator to remotely induce a rapid heating performance.

**Fig. 4:**
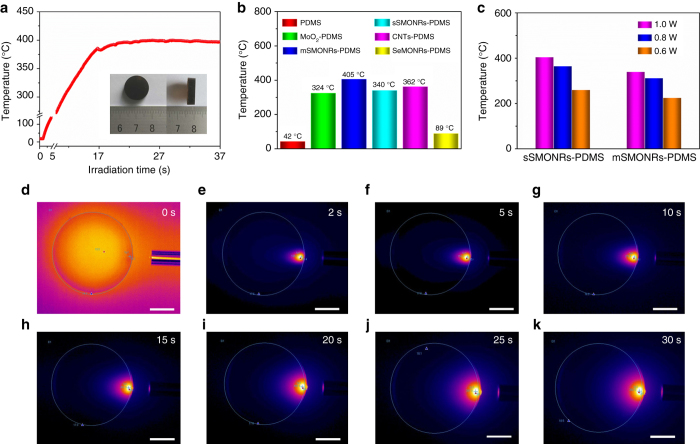
Photothermal performance of the sSMONRs-PDMS composite. **a** Temperature of the sSMONRs-PDMS composite upon NIR laser irradiation; an 808-nm laser was used with a power density of 1 W/cm^2^; the inset in (a) shows a photograph of the sSMONRs-PDMS composite; the data discontinuity was caused by a variation of the temperature measurement range; **b** chart showing the temperature of the various composite (the temperature value is defined as maximum equilibrium temperature upon irradiation); **c** chart showing the photothermal performance of the sSMONRs-PDMS composite and mSMONRs-PDMS composite under different irradiation powers; **d**–**k** the monitored photothermal images of the sSMONRs-PDMS composite recorded by an online thermocouple thermometer (the top right values represent the irradiation times). All scale bars are 5 mm

**Fig. 5:**
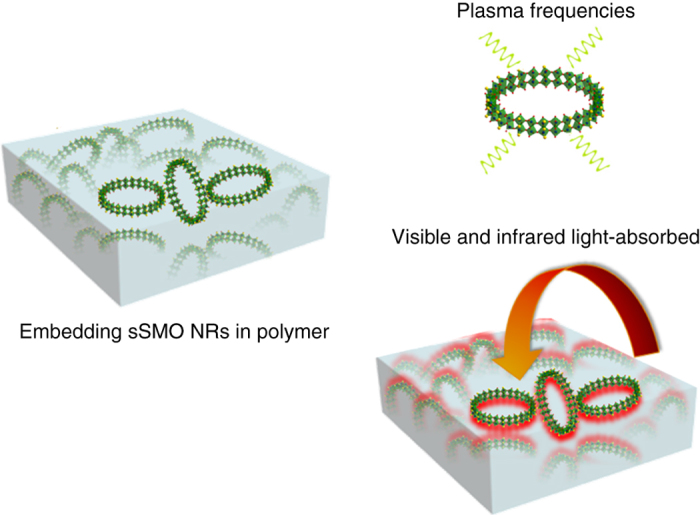
Schematic of the light-induced photothermal effect of the sSMO NRs in the polymer. Upon light irradiation, the strong light absorption of the sSMO NRs quickly and efficiently converts light energy into heat in the polymer

Given that the sSMO NRs possess an all-optical absorption and great compatibility with the polymer, we designed here a sSMO NRs-based polymer composite and investigated its light-induced photothermal effect. First, we introduced the sSMO NRs into a polymer to obtain a sSMONRs-PDMS composite (Supplementary Fig. 18). To measure the photoinduced heating effect, an online-type thermocouple thermometer was employed to record in situ the temperature change process. As shown in Fig. 4a, the temperature dramatically increased under the irradiation of an 808-nm lasers (Intensity: 1 W/cm^2^), generating the local temperature up to 400 °C within 20 s, which is the highest temperature by light irradiation to date. Moreover, PDMS showed a negligible temperature rise with infrared laser irradiation over several minutes (Supplementary Fig. 19)^41, 42^. We also compared the photothermal performance of various composites (Fig. 4b) and found that the sMONRs-PDMS composite exhibited the best photothermal performance (Supplementary Fig. 20), which is attributed to the strong NIR-light absorption and excellent compatibility with the polymer. In addition, the sSMONRs-PDMS composite showed a higher photothermal performance than that of the mSMONRs-PDMS composite under the same power density (Fig. 4c). Figure 4d–k shows the consecutive photothermal images under continous irradiation. A sharp increase in temperature occurred during the initial stages, attributed to the strong NIR-light absorption, followed by a relative heat equilibrium state. The extremely high temperature is attributed to the strong infrared-light absorption and the heat accumulation in the thermal insulator polymer. As the laser is switched off, the temperature decreases rapidly. Notably, the extremely high temperature generates an obvious ablation and smoking phenomenon under NIR-laser irradiation (Supplementary Movie 3). After switching the NIR laser on and off several times, a negligible temperature reduction was observed (Supplementary Fig. 21), which is ascribed to the structural damage and aggregation of the NRs under high temperature (Supplementary Fig. 22). As shown in Fig. 5, the strong light absorption allowed the sSMO NRs to quickly and efficiently convert incident light energy into heat, leading to a rapid heating performance. In addition, the temperature is closely related to the laser power density and irradiation distance. When the laser power density decreased to 0.8 and 0.6 W cm^2^, high temperatures of  ~ 365 and 260 °C, respectively, remained under irradiation (Supplementary Fig. 23). Under the same laser power density, short radiation distances resulted in higher temperatures (Supplementary Fig. 24). Therefore, the temperature was closely related to the laser power density and irradiation distance. Such impressive heating behavior indicates that the sSMO NRs can be employed as a photothermal transduction agent to remotely induce a photoresponse.

**Fig. 6:**
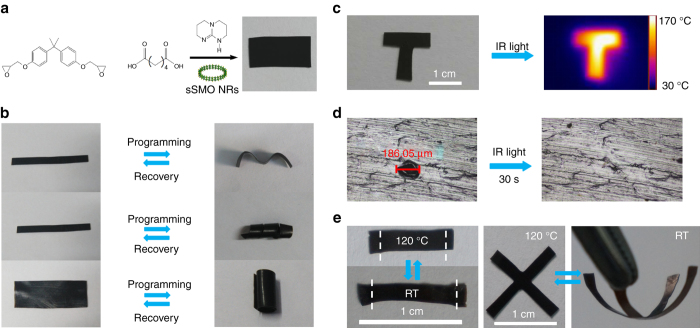
Light-activated performance of the sSMONRs-epoxy composite. **a** Synthesis of sSMONRs-virimer; **b** demonstration of the shape memory effect of sSMONRs-virimer under visible light; **c** photothermal image of sSMONRs-virimer; photothermal image of the letter T under IR irradiation (808 nm, 1.0 W/cm^2^); **d** self-healing measurements under IR irradiation; **e** reversible actuation and dynamic structures of sSMONRs-xLECs by IR irradiation

As discussed above, the all-optical absorption of the sSMO NRs allows us to investigate their visible-light-response property. Herein, as shown in Fig. 6a, the sSMO NRs embedded in a thermo-responsive vitrimer were fabricated according to our previous method^43^. The sSMONRs-vitrimers composite (3 wt%) was exposed to visible light to investigate its light-response properties (Supplementary Fig. 25). Figure 6b gives an example of the shape memory effects of the sSMONRs-vitrimer composite. A sSMONRs-vitrimer strip can be easily reconfigured into various bending or overlaped geometries. Under visible-light irradiation, these temporary shapes were quickly restored to their original strip shape in seconds (Supplementary Fig. 26 and Supplementary Movie 2), indicating an excellent visible-light shape memory response. The temperature of the sSMONRs-vitrimer composite quickly increased to  ~ 70 °C under visible-light irradiation with an intensity of 140 mW/cm^2^, which is higher than the glass-transition temperature (*T*_g_) of the sSMONRs-vitrimer (Supplementary Fig. 27), endowing the material with visible-light-activated shape memory. Even with a reduced power of 50 mW/cm^2^ (Supplementary Fig. 28), the bending structure was still restored to the original structure upon an increased time.

The self-healing and reshaping performance were investigated upon exposure of infrared laser irradiation. As shown in Fig. 6c, the temperature of the sSMONRs-vitrimer composite increased to  ~ 170 °C, owing to the strong NIR absorption behavior, which exceeds the topology-freezing transition temperature (*T*_v_). As the temperature increases above *T*_v_, transesterifcation reactions can be triggered (Supplementary Fig. 29)^44^. Here, localized transesterification reactions were initiated by light-induced heating, allowing a local damaged area to automatically self-heal. As shown in Fig. 6d, a typical 186-μm wide hole was in situ self-healed in 30 s (intensity: 1.47 W/cm^2^) without the help of any healing agent, whereas a wider crack of 228 μm was still healed with a slightly longer irradiation time (Supplementary Fig. 30). For comparison, no healing was observed for the pure vitrimer without adding the sSMO NRs under the same light intensity. Meanwhile, reshaping of the sSMONRs-vitrimer composite was also achieved under continuous irradiation (Supplementary Fig. 29b). Therefore, we demonstrated that the excellent photothermal conversion of the sSMO NRs provide a quick increase of the local temperature and remotely triggered fast transesterification, leading to self-healing of microcracks and shape deformations.

To achieve controlled manipulations such as reversible actuation and dynamic three-dimensional (3D) changes, we selected xLCEs as a polymer matrix owing to its high mechanical strength and moldable properties^45^. The sSMONRs-xLCEs composite was fabricated by terpolymerization of diglycidyl ether with 4,4′-dihydroxybiphenol and sebacic acid in the presence of triazobicyclodecene (Supplementary Fig. 31a)^46^. On the basis of the differential scanning calorimetry results (Supplementary Fig. 31b), the glass-transition temperature (*T*_g_) and isotropic-transition temperature (*T*_*i*_) of sSMONRs-xLCEs were  ~ 52 and 110 °C, respectively. Remarkably, the aligned film can reversibly changed length between the liquid crystalline phase and the isotropic phase (110 °C). This aligned sSMONRs-xLCEs film can reversibly changed, when cooled down to below T_i_ and shrunk when reheated to above *T*_i_ (Supplementary Fig. 31c). Moreover, as shown in Fig. 6e, the extent of the alignment modes of sSMONRs-xLCEs could be programmed by manipulating the irradiation energy, resulting in a temperature gradient along the composite to realize a dynamic 3D structures (Supplementary Fig. 32). In addition, more interesting triple shape memory performance was achieved with appropriate manipulation (Supplementary Fig. 33). All the above results have clearly demonstrated that by dispersing the sSMO NRs into a polymer enables to design and build dynamic 3D structures upon infrared laser irradiation.

## Discussion

To summarize, we demonstrated a simple, large-scalable method to prepare free-standing SMO NRs with an atomic-level size and tuneable ring-in-ring architecture. The size of the SMO NRs was only 0.5 nm, which is equal to the thickness of 2–3 atomic layers. The key contributors to the ring-in-ring structure was the sulfur doping and the surface–ligand interactions. Owing to the amorphous and ultrathin structure, the resulting sSMO NRs exhibited strong visible and infrared absorption properties. As a result, the sSMO NRs can function as a highly efficient light-heat conversion agent. As a proof of concept, a composite of the sSMO NRs filled with PDMS exhibited an ultrafast and impressive photothermal effect upon NIR laser irradiation. Most importantly, a light-driven composite was designed by dispersing the NRs into vitrimer, allowing for localized transesterification to be remotely triggered, resulting in visible-light-activated shape memory. Meanwhile, self-healing and shape-deformation performances were achieved upon exposure to NIR laser irradiation. Moreover, such a light-induced method allows to control the alignment of the xLCEs, leading to a reversible actuation of dynamic 3D structures. The photothermal effect demonstrated here is not restricted to the current system and can be incorporated into electronic and optical devices. We believe that the present finding demonstrates the advancement of atomic-level nanostructures and opens exciting opportunities for broad applications in catalysis, optics and optoelectronics.

## Methods

### Chemicals

Ammonium heptamolybdate [(NH_4_)_6_Mo_7_O_24_·4H_2_O], thiourea and oleyl alcohol (OA) were purchased from Sinopharm Chemical Reagent Beijing Co., Ltd. Oleylamine (OM) was purchased from J&K Scientific. Deionized water was used and all the chemicals were used as received. Diglycidyl ether of bisphenol A (DGEBA, Sigma-aldrich, DER 332), adipic acid (TCI > 99.0%), Triazobicyclodecene (TCI 98%), Triazobicyclodecene (Sigma-aldrich 98%) and sebacic acid (Aladdin, CP) were used directly without further purification.

### Synthesis of atomic-level mSMO NRs and sSMO NRs

In a typical synthesis, ammonium heptamolybdate ([(NH_4_)_6_Mo_7_O_24_·4H_2_O]) and thiourea with a mole ratio of 3.5:0.5 were dissolved in 5 ml water. Then 2 ml OM and 4 ml OA were added to the aqueous solution with vigorous stirring, forming a homogeneous solution. The autoclave was sealed and heated at 200 °C for 6 h in an oven. After cooling down to room temperature, the product was collected by centrifugation and washed several times with ethanol. For the synthises of sSMO NRs, sSMO NRs can be obtained by increasing the amounts of thiourea (3.5:1) while keeping other constant.

### Synthesis of MoO_2_ NPs and oxygen-incorporated MoS_2_ nanosheets

In a typical synthesis, a certain amounts of ammonium heptamolybdate ([(NH_4_)_6_Mo_7_O_24_·4H_2_O]) was dissolved in 2 ml water. Then 2 ml OM and 5 ml OA were added to the aqueous solution with vigorous stirring, forming a homogeneous solution. The autoclave was sealed and heated at 200 °C for 6 h in an oven. After cooling, the product was collected by centrifugation and washed several times with ethanol. Oxygen-incorporated MoS_2_ nanosheets can be obtained by adding the excess thiourea, while maintaining others conditions the same as nanorings. All of the products are denoted as S_*x*_, for convenience, where x represents the precursor Mo/S molar ratios. For example, the sample with molar ratios of Mo to S (3.5:10) denoted as S_10_.

### Synthesis of atomic-level SeMO NRs

In a typical synthesis, ammonium heptamolybdate ([(NH_4_)_6_Mo_7_O_24_·4H_2_O], 300 mg) and selenium powder (20 mg) were continuously added in 1 ml water under ultrasonic treatment. Then 3 ml OM and 2 ml OA were added to the aqueous solution with vigorous stirring, forming a homogeneous solution. The autoclave was sealed and heated at 200 °C for 6 h in an oven. After cooling down to room temperature, the product was collected by centrifugation and washed several times with ethanol for further characterization.

### Preparation of sSMONRs-PDMS composite and mSMONRs-PDMS composite

PDMS polymer was fabricated according to the previous publications. In a typical synthesis, a 10:1:1 (wt:wt:wt) mixture of Sylgard-184 silicone elastomer base (Dow Corning Corp., Midland, MI), silicone elastomer curing agent, and a cyclohexane solution with sSMO NRs or mSMO NRs (about 6.5 mmol) were thoroughly mixed in a glass container. After degassing by ultrasonic treatment, the liquid mixture was aged at 30 °C for 10 h and cured at 90 °C for 1 h in a vacuum oven.

### Preparation of sSMONRs-vitrimer

As a typical example, stoichiometric amount of diglycidyl ether of bisphenol A (0.340 g, 1.0 mmol) and adipic acid (0.142 g, 1.0 mmol) were mixed with 4 ml chloroform dispersion (SMO NRs, 3 wt%) under ultrasonic treatment for 30 min. Then the mixture was heated to 100 °C till the chloroform was totally evaporated and then the mixture was heated to 180 °C and melted. Then, triazobicyclodecene (5 mol% to the COOH) groups was introduced and stirred manually till homogeneous. After cooling down to room temperature, the mixture was sandwiched between two glass slides. The two glass slides with the reaction mixture in the middle were clamped together and left at 180 °C for at least 4 h. The thickness of sSMONRs-vitrimer films can be changed by controlling the spacer of between the two plates with 6 MPa pressure.

### Preparation of sSMONRs-xLCEs

sSMONRs-xLCEs can be obtained by reacting diglycidyl ether of 4, 4′-dihydroxybiphenol with sebacic acid, using triazobicyclodecene as a transesterification catalyst. Diglycidyl ether of 4, 4-dihydroxybiphenol (DGE-DHBP) was synthesized according to the previous method^47^. As a typical experiment, stoichiometric amount of DGE-DHBP (0.298 g, 1.0 mmol) and sebacic acid (0.202 g, 1.0 mmol) were mixed with 4.0 ml sSMONRs dispersion obtained from the above procedure and sonicated for 1 h. Afterwards, the mixture was heated to 100 °C till the chloroform was totally evaporated and then the mixture was heated to 180 °C and melted. Then triazobicyclodecene (13.9 mg) was subsequently introduced and stirred manually to get a homogenous mixture. As the mixture became very viscous, it was then cooled down to room temperature to afford a solid product that was not completely crosslinked. Then the mixture was sandwiched between two plates to be cured by a hot press for 4 h at 180 °C. The thickness of sSMONRs-xLCEs films can be changed by controlling the spacer between the two plates with 6 MPa pressure.

### Characterization

The morphology and size of the samles were characterized by a HITACHI H-7700 TEM with an accelerating voltage of 100 kV, and a FEI Tecnai G2 F20 S-Twin high-resolution TEM equipped with energy dispersive spectrometer (EDS) analyses at 200 KV. The scanning electron microscope (SEM) was performed on a LEO 1530. The crystal structure was determined by XRD on a Rigaku D/max-2400 X-ray diffractometer using CuK radiation (λ = 1.5418 Å) at 40 kV voltage and a 40 mA current ranging from 10° to 80°. X-ray photoelectron spectroscopy (XPS) experiments were carried out on scanning X-ray microprobe (Quantera SXM, ULVAC-PHI. INC) operated at 250 kV, 55 eV with monochromated Al Kα radiation. The UV–vis absorption spectrum were obtained using a Shimadzu UV-3600 spectrometer. The thermal stability of sample was measured with a TA-Q50 thermal gravity analysis (TGA) under air atmosphere. Differential scanning calorimetry (DSC) measurements were performed by TA-Q2000 at a scanning rate of 10 °C/min. To measure the photothermal conversion performance of the samples, an 808 nm NIR laser with an external adjustable power (1 W/cm^2^) and a 5 mm diameter laser module was used to excite the samples through a quartz cuvette containing a dispersion (1 ml) of the samples. And the temperature was recorded by an online-type thermocouple thermometer with an accuracy of ± 0.1 °C.

### XAS analysis

Mo K-edge XAFS measurements were conducted at the beamline 14W1 in Shanghai Synchrotron Radiation Facility (SSRF). The X-ray was monochromatized by a double-crystal Si (111) monochromator both for SSRF and BSRF, and the energy was calibrated using a molybdenum metal foil. The monochromator was detuned to reject higher harmonics. The acquired EXAFS data were processed according to the standard prcedures using the WinXAS3.1 program^48^. Theoretical amplitudes and phase-shift functions were calculated with the FEFF 8.2 code^49^ using the crystal structural parameters of the MoO_2_ and MoO_3_ foil.

### Photothermal heating of sSMONRs-PDMS composite

To study the photothermal heating of hybrid composites, embedding nanorings in PDMS composite were irradiated by a NIR laser (808 nm, 1 W/cm^2^). The temperature and thermal imaging picture were recorded by an online-type thermocouple thermometer. Noted that the recorded temperature is the local maximum temperature of the composite. The distance between laser launcher and composite is 0.5 cm.

### Photothermal conversion effect

In order to study the photothermal effect, various samples dispersed in cyclohexane (1 ml) were irradiated by continuous NIR irradiation (808 nm) a 5 mm diameter laser module under a power density of 1.0 W/cm^2^. Upon NIR laser irradition, the temperature was recorded by an online-type thermocouple thermometer.

### Photo-responsive reshaping of sSMONRs-vitrimer composite

To reshape the sample by light, a sSMONRs-vitrimer film (15.32 mm × 1.62 mm × 0.20 mm) was irradition at the ridge under an intensity of 1.47 W/cm^2^ for 2 min, while the straight strip was bent into a specific shape through the external force. As external stress was removed, it was found that the specific shape did not change.

### Photo-responsive healing of sSMONRs-vitrimer composite

To verify the self-healing of sSMONRs-vitrimer, a special hole was first carved with a razor to get a cut of about 186 mm in width. Under the irradiation of NIR laser with an 1.47 W/cm^2^. The hole can be well healed after irradiation within a minute.

### Reversible actuation of sSMONRs-xLCEs composite

To verify the reversible thermal actuation of sSMONRs-xLCEs, An aligned sSMONRs-xLCEs strip was irradiation by an intensity of 1.47 W/cm^2^, the lengthe of sSMONRs-xLCEs can be stretched when cooled down to below room temperature and shrinked when reheated to above at high temperature (*T*_*i*_).

### Dynamic 3D structures of sSMONRs-xLCEs composite

The sSMONRs-xLCEs were first processed into desired shape. Each petal was stretched and exposed to light irridation one by one under an intensity of 1 W/cm^2^, making a temperature gradient along the thickness the film, leading to the reversible bending movement.

### Data availability

The relevant data that support the findings of this study are available from the authors upon reasonable request.

## Electronic supplementary material

Supplementary Information

Description of Additional Supplementary Files

Supplementary Movie 1

Supplementary Movie 2
