# Supplementary material for: Atomic-level molybdenum oxide nanorings with full-spectrum absorption and photoresponsive properties
Source: Nat Commun. 2017 Nov 16;8:1559. doi: 10.1038/s41467-017-00850-8 (PMC5691127; doi:10.1038/s41467-017-00850-8)
Supplement: Supplementary file 1 — Supplementary Information [file 41467_2017_850_MOESM1_ESM.pdf]

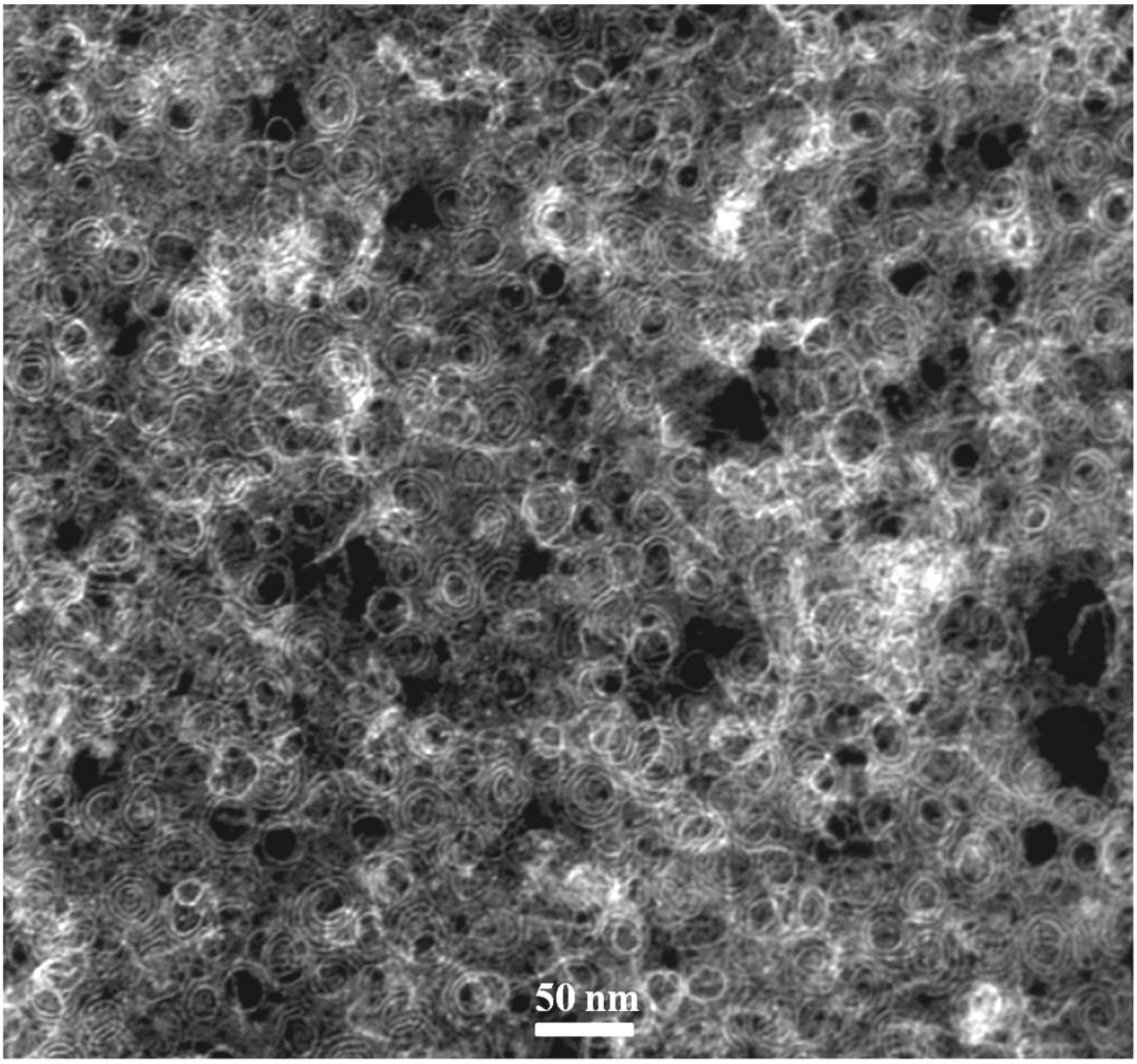

Supplementary Figure 1. Annular dark-field STEM image of as-synthesized mSMO NRs.

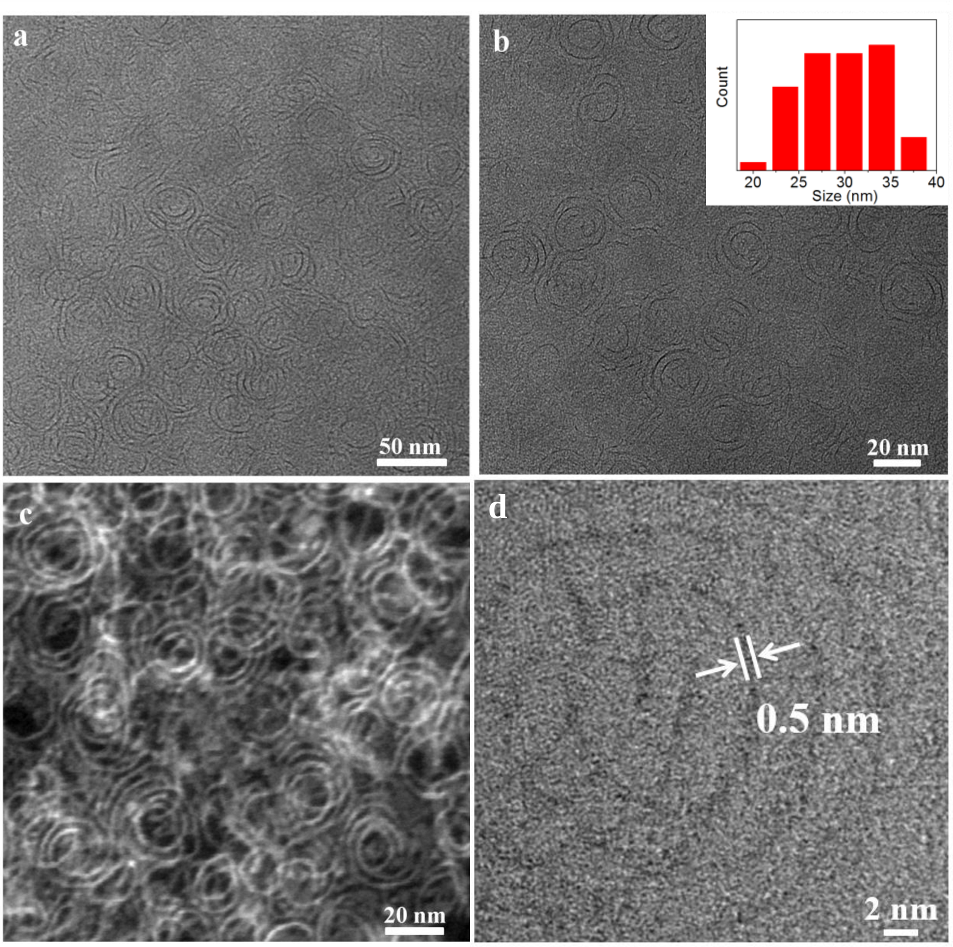

Supplementary Figure 2. (**a-b**) TEM images of as-prepared mSMO NRs under different magnifications; The inset in (b) is the size distribution histogram of the outermost layer NRs, showing a size distribution of about 30 nm; (**c**) STEM images of mSMO NRs under different magnifications; (**d**) HRTEM image of mSMO NRs. Noted that the thickness of NRs is only 0.5 nm.

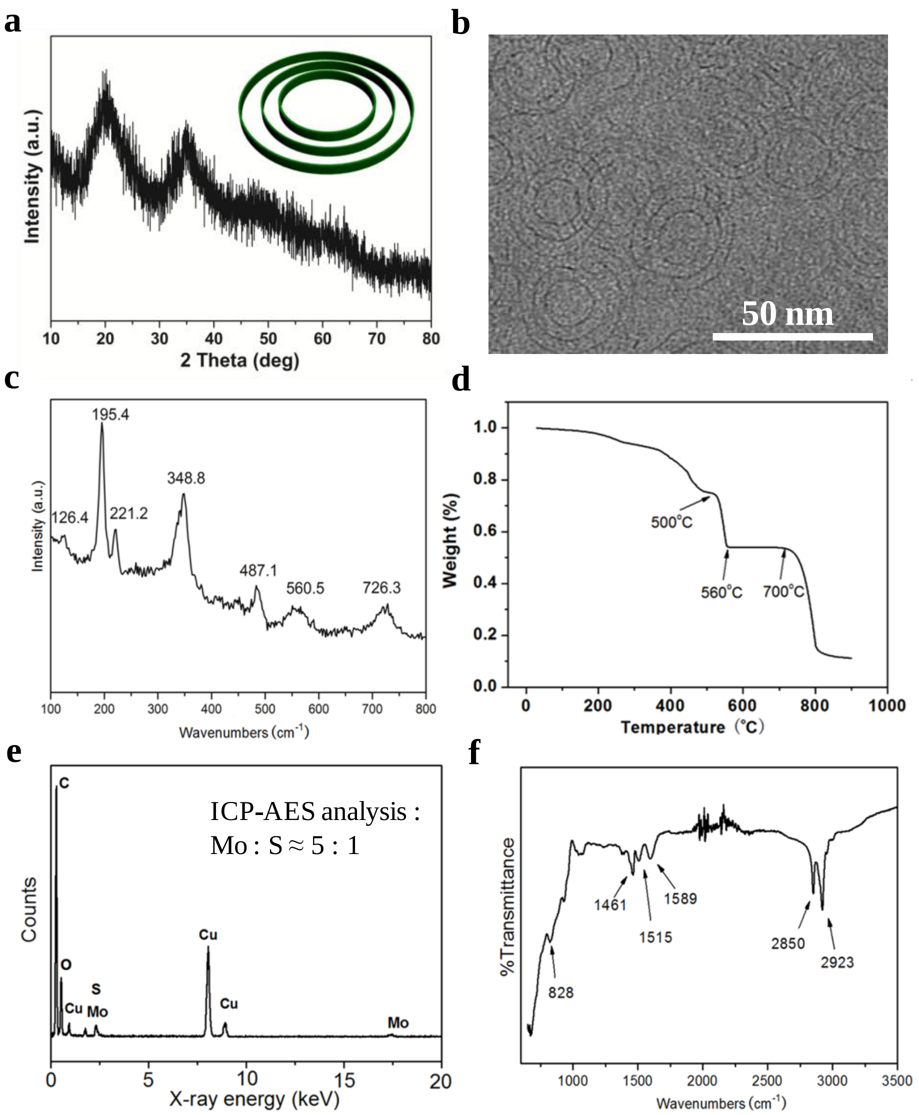

Supplementary Figure 3. (a-b) XRD pattern and TEM image of the mSMO NRs. The inset is the structural model of ring-in-ring structure; (c) Raman spectrum of as-prepared ultrathin mSMO NRs; (d) TGA curve of as-prepared mSMO NRs; (e) EDX spectrum of mSMO NRs, indicating the existence of Mo, S and O element; The atomic ratio of Mo: S is about 5: 1 based on the ICP-AES analysis. (f) FT-IR spectra of mSMO NRs.

In the Raman spectrum, the signal at 560.5 and 726.3 cm^{-1} are characteristic for the telescopic vibration mode of molybdenum oxide. The rest of the peaks including at 126.4, 195.4, 221.2, 348.8 and 487.1 cm^{-1} show a litter shift from the standard values of MoO_2 sample, which may be ascribed to the amorphous structure caused by sulfur doping. There is a sharp decrease between 500 °C to 700 °C due to the decomposition of organic surfacant and a sharp drop at the sublimation temperature of molybdenium oxides around 700 °C. This phonemenon was consist with the molybdenum dioxide compounds. Noted that the peaks centered at 1450-1600 cm^{-1} and 2800-3000 cm^{-1} were assigned to N-H modes and he CH_2 and CH_3 stretching vibrations, indicating that the sample was capped with octylamine. In addition, the stretching vibrations of Mo=O bonds and Mo-O-Mo skeleton were exhibited by the peaks at 1384 and 400 cm^{-1}. Based on the mentioned above, the structure of the NRs can be considered as S-doped molybdenum dioxide.

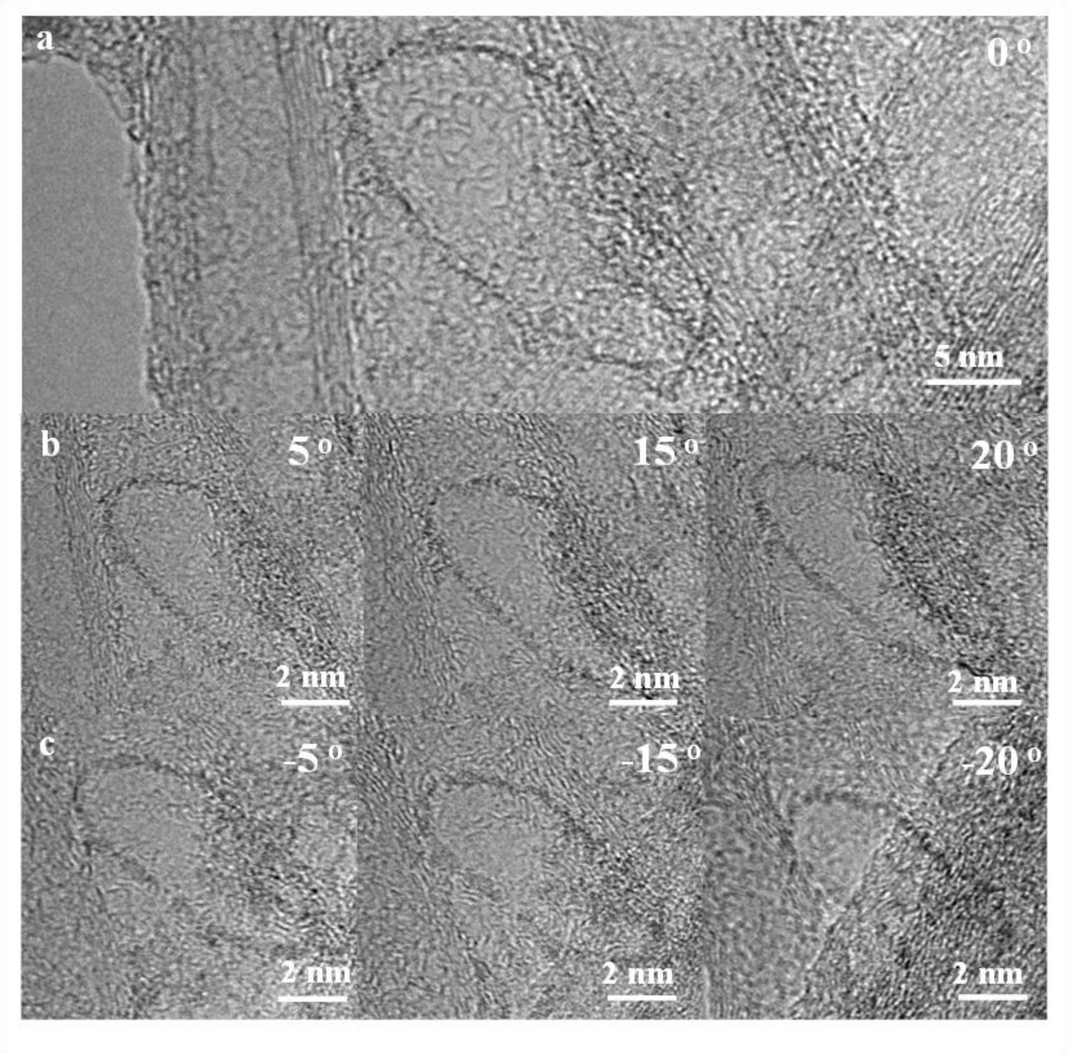

Supplementary Figure 4. (a-c) A series of tilted HRTEM images of NRs from -20° to +20°.

In order to show the side structure of NRs, the NRs was loaded on a carbon nanotubes grid. According to HRTEM images, there was no visible lattice fringes for NRs, further indicating the amorphous feature. Based on a series of tilted HRTEM images, the side structure of NRs can be clearly seen and the thickness of NRs is about 0.5 nm. With a tilt angle of +20°, NRs exhibits almost vertical status. Under beam irradiation, the structure of the ring is unstable, and finally destroyed under longer irradiation time.

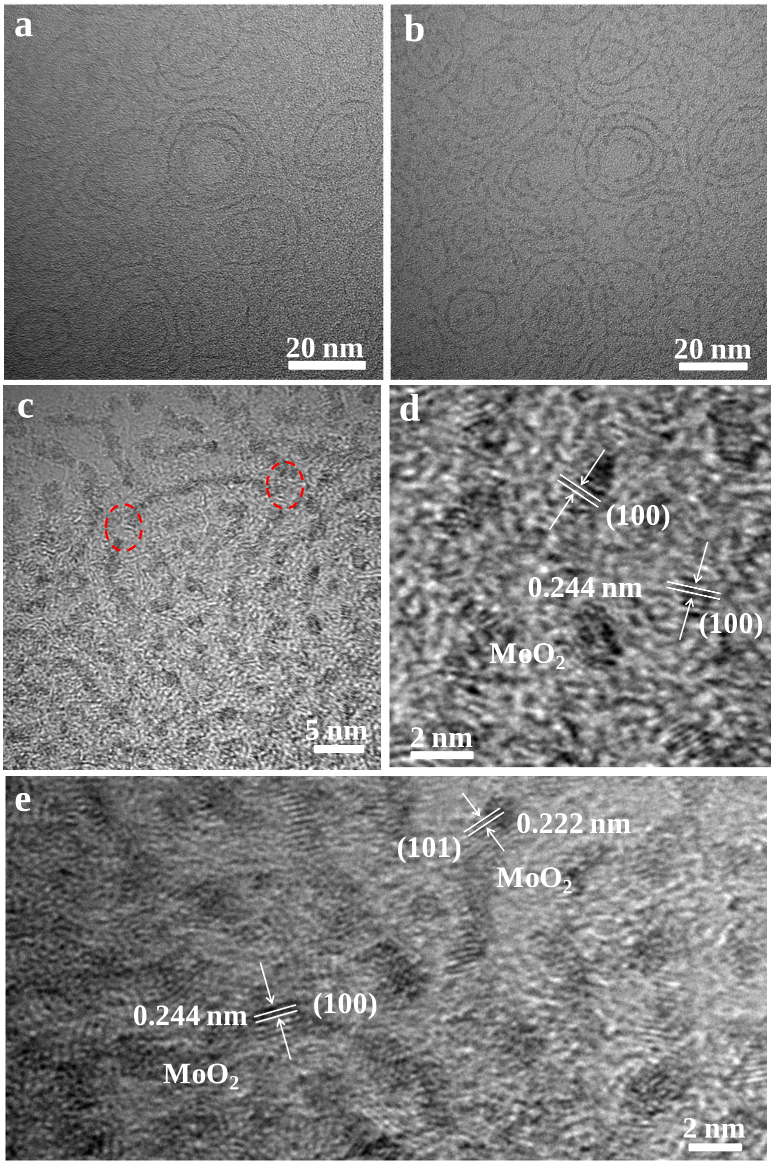

Supplementary Figure 5. (a-b) Consecutive HRTEM snapshots of the breaking and recrystallization process of mSMO NRs under beam irradiation; **(c)** TEM images of seperated nanostructure under irradiation; **(d-e)** HRTEM images of well-crystallized nanostructure after baem irradiation.

The as-obtained atomic-level mSMO NRs is electron beam sensitive, which is illustrated by the observation of electron-beam-induced damage and crystallization. Under beam irraditon, it gradually split from one defect point and shrunk into two parts. Obvious lattice fringes can be detected, undergoing a clear amorphous to crystalline transformation. The lattice spacing of 0.222 nm and 0.244 nm correspond to the (101) plane and the (100) plane of MnO_2.

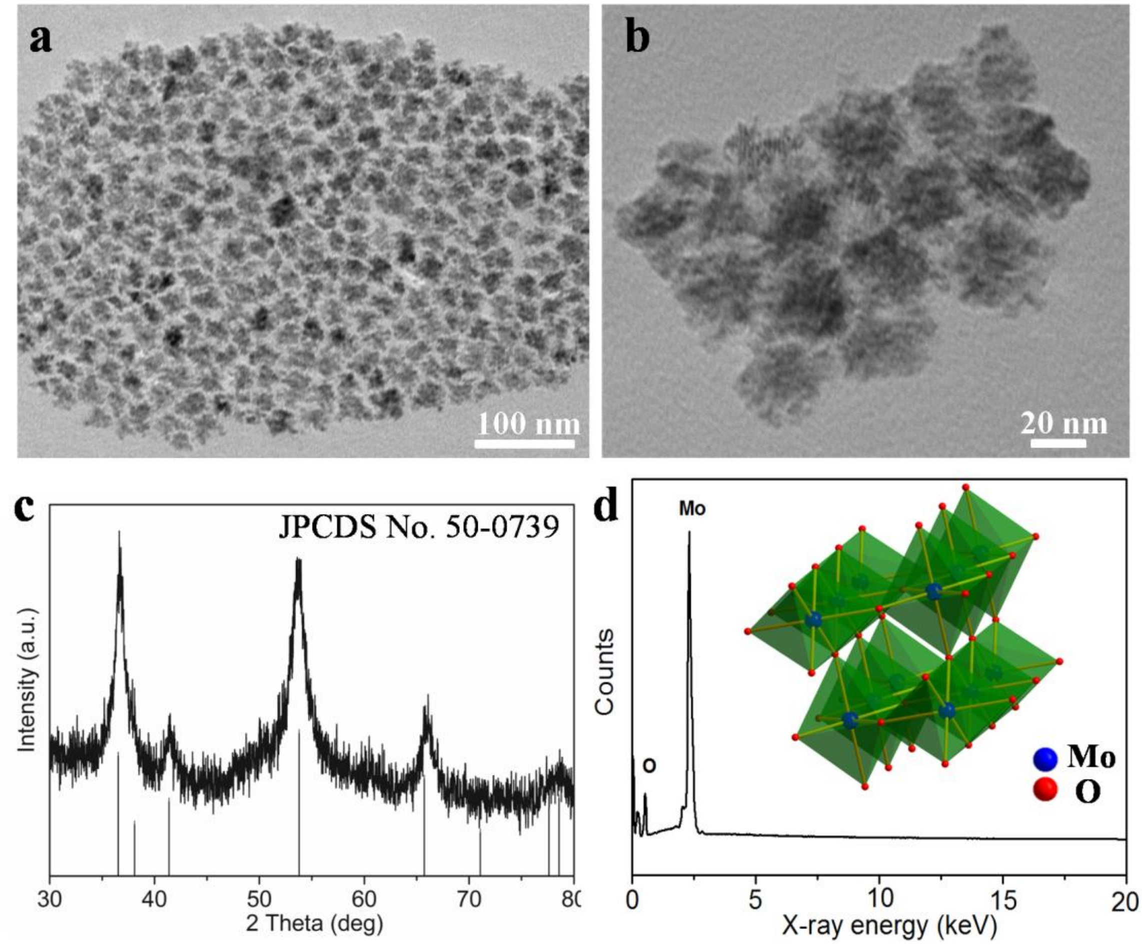

Supplementary Figure 6. (**a-b**) TEM images of MoO_2 NPs; (**c**) XRD pattern of MoO_2 NPs, indicating the formation of hexagonal MoO_2 phase; (**d**) Energy-dispersive X-ray spectrums of MoO_2 NPs. Inset: Structure model of hexagonal MoO_2 structure;

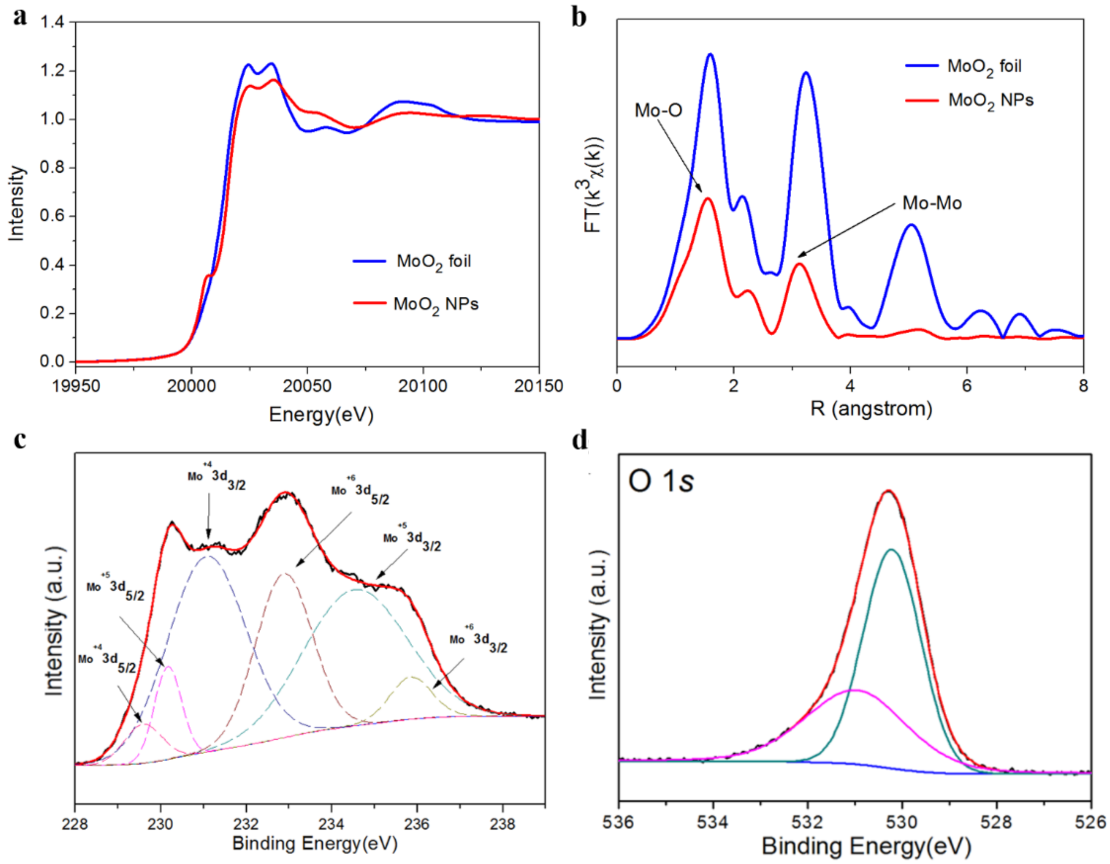

Supplementary Figure 7. (**a**) XANES patterns and (**b**) Fourier transforms of the Mo K-edge EXAFS patterns of MoO_2 NPs, and MoO_2 foil; (**c-d**) XPS spectra of Mo 3*d* and O 1*s* peak for MoO_2 NPs.

For MoO_2 NPs, Mo K-edge X-ray absorption near edge structure (XANES) spectrum indicate that the energy positions, the shape, and intensity of the absorption edges were almost similar with those of the MoO_2 reference compound. The preedge feature observed in the XANES spectra show that the valence states of elements Mo is slightly higher than MoO_2 foil. Furthermore, Mo K-edge extended X-ray absorption fine structure (EXAFS) spectra show that the obtained MoO_2 NPs possess many oxygen vacancies because the coordination number for the first shell is 3.9 (Supplementary Table 2).

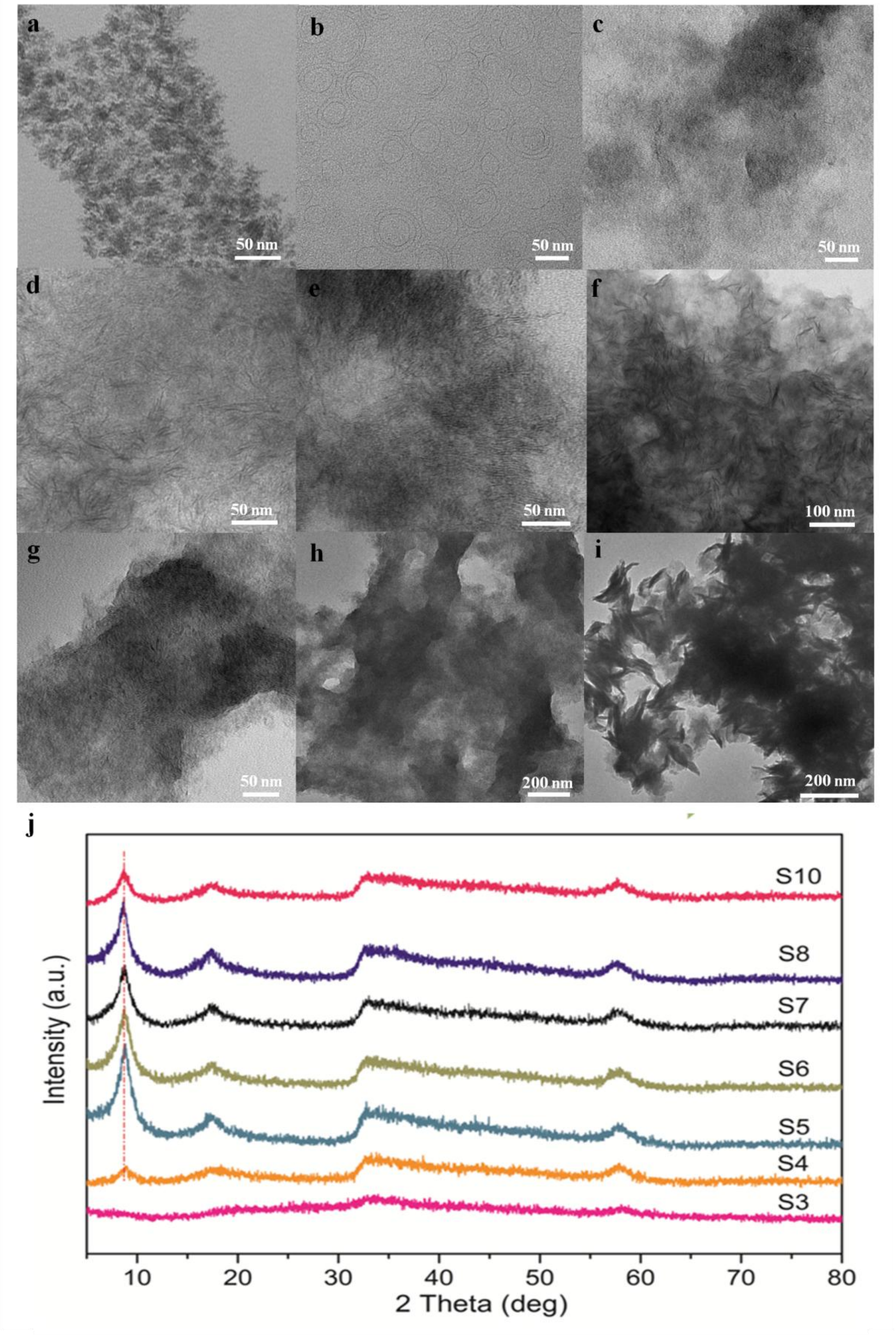

Supplementary Figure 8. TEM images of the samples with different ratios of Mo/S precursor. **(a)** MoO_2 NPs; **(b)** mSMO NRs; **(c)** S2; **(d)** S3; **(e)** S4; **(f)** S5; **(g)** S7; **(h)** S8; (**i**) S10; **(j)** The corresponding XRD patterns of the samples. With increasing sulfur content, the morphology evolves from nanoparticle to nanrings, finally to nanosheets. Noted that one new peak near $2\theta=10^o$ with a sharp peak represents the spacings of between adjacent planes about 9 Å.

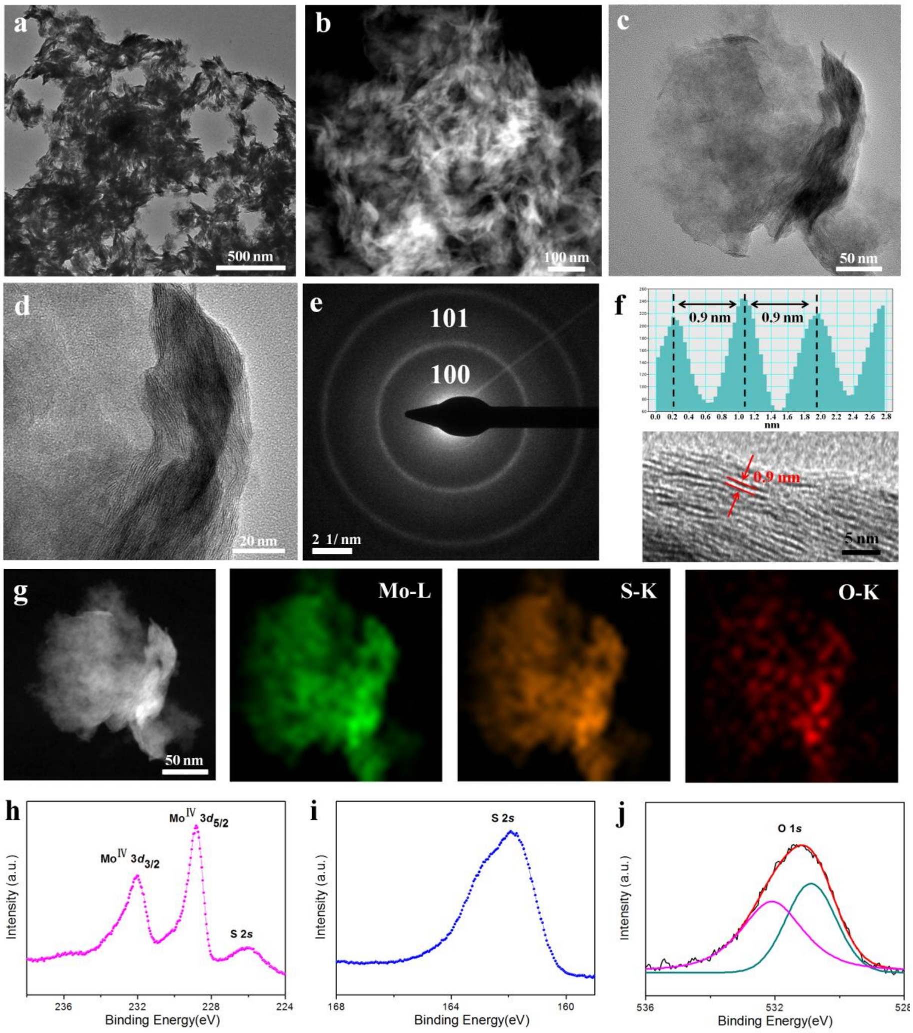

Supplementary Figure 9. (**a-d**) STEM and TEM images of the sample; (**e**) SAED pattern of the sample; (**f**) HRTEM image of the sample. Inset: profile plot of the calibration for measuring the spacings; (**g**) HAADF-STEM image and the corresponding EDS mapping images, indicating the homogeneous distribution of S, Mo and O element; (**h-j**) XPS spectra of Mo 3*d*, S 2*s* and O 1*s* peaks for the oxygen-incorporated MoS_2 nanosheets.

It was clearly see that the product (S10) consists of many curly nanosheets. The typical SAED pattern shows concentric diffraction rings that can be indexed to the (100) and (110) diffractions of MoS_2. Interestingly, an interlayer spacing of nanosheets is approximately 0.9 nm, which is greater than the (002) spacing of hexagonal 2H-MoS_2 (0.65 nm). This phenomenon is similar with the previous oxygen-incorporated MoS_2. The EDS elemental mapping result further confirmed the exist of oxygen. The binding energies of Mo 3*d* 3/2 and Mo 3*d* 5/2, peaks are located at 232 and 228.8 eV, respectively. The binding energies can be attributed to $Mo^{(IV)}$ in the product. The binding energies at 161.7 eV in S 2*S* spectra are characteristic of the S^{2-} of MoS_2. Based on the results mentioned above, the oxygen-incorporated MoS_2 nanosheets were prepared under the condition of excess thiourea.

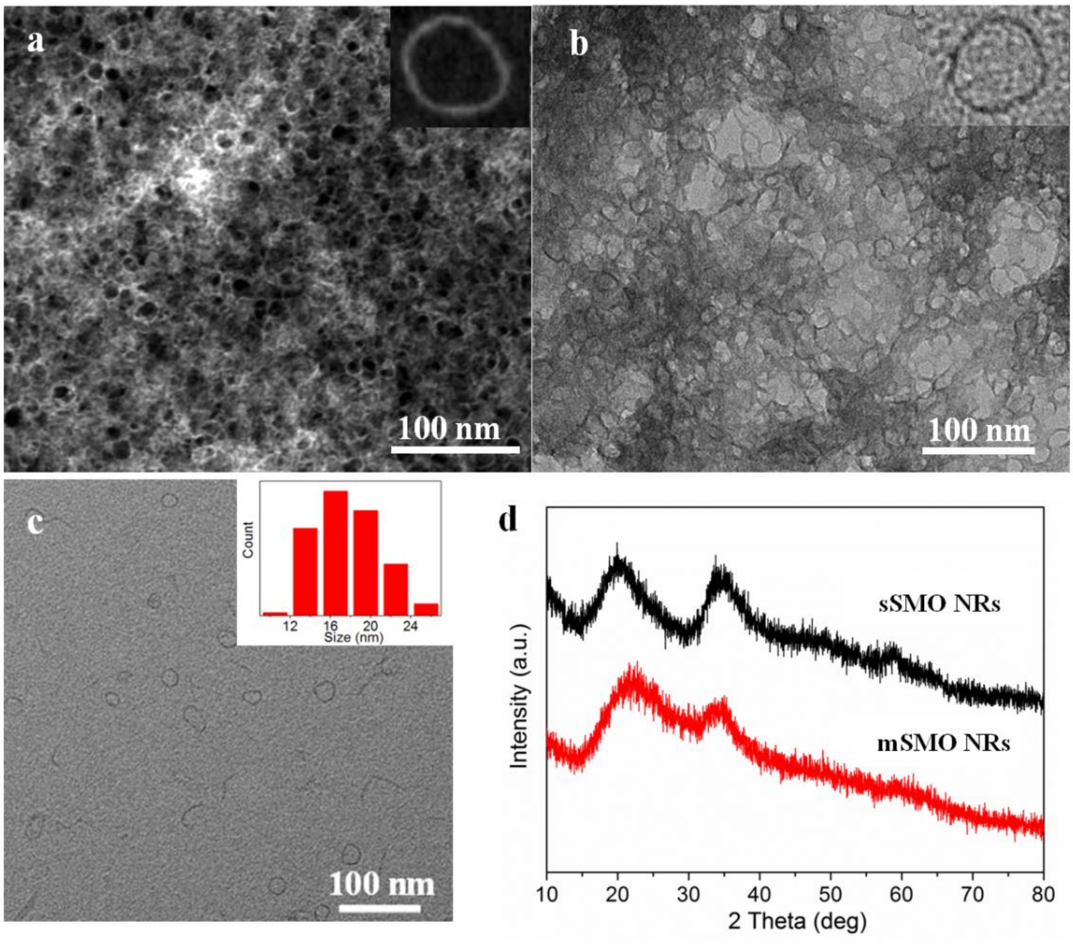

Supplementary Figure 10. (a-b) STEM and TEM images of the sSMO NRs. The inset is the typical image of single NRs; **(c)** TEM images of the sSMO NRs. The inset is the size distribution histogram of the sSMO NRs, showing a size distribution of about 18 nm; **(d)** XRD patterns of the mSMO and sSMO NRs.

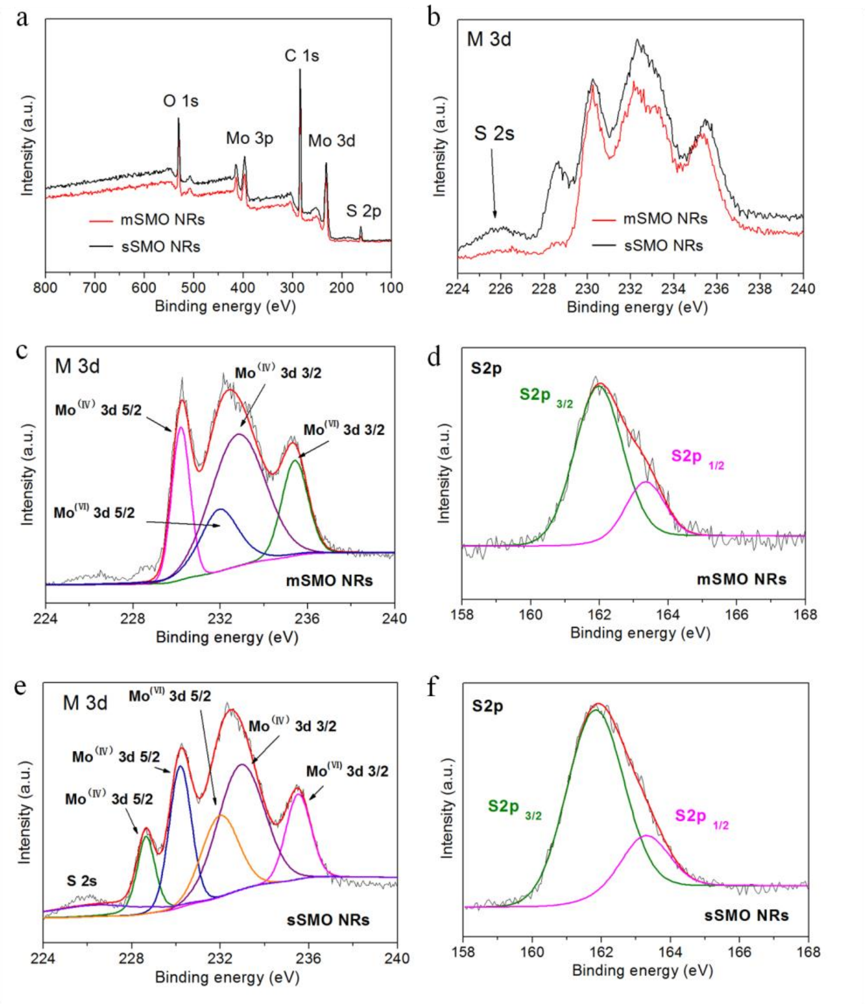

Supplementary Figure 11. (a) XPS spectra of mSMO NRs and sSMO NRs; **(b)** Mo 3d spectrum of mSMO NRs and sSMO NRs; **(c-d)** XPS spectrum details for Mo 3d binding energy regions and S 2s level for mSMO NRs; **(e-f)** XPS spectrum details for Mo 3d binding energy regions and S 2s level for sSMO NRs.

The atomic valence states of the composition of mSMO NRs and sSMO NRs were characterized by XPS. According to the survey spectra, the elements of Mo, S, O and C can be clearly identified. With the increasing of sulphur doping, there was obvious difference in the atomic valence states of the NRs. Compared with the high-resolution XPS of the Mo 3d regions of mSMO NRs, the varieties of the peaks was more for sSMO NRs, indicating the complicated electronic structure. The Mo 3d spectrum of mSMO NRs can be divided into four peaks. The binding energy peaks of Mo 3d 5/2 (231.9 eV) and Mo 3d 3/2 (235.4 eV) are characteristics of MoO_3, while two peaks of Mo 3d 5/2 (230.18 eV) and Mo 3d 3/2 (232.8 eV) components are characteristic of MoO_2. The presence of MoO_3 origiates from the inevitably surface oxidation. For sSMO NRs, the Mo 3d spectrum of

mSMO NRs can be divided into five peaks. The binding energy peaks of Mo 3d 5/2 (231.9 eV) and Mo 3d 3/2 (235.2 eV) are characteristics of MoO_3. Actually, two peaks of Mo 3d 5/2 (230.1 eV) and Mo 3d 3/2 (232.9 eV) components are characteristic of MoO_2. Notably, the presence of the shoulder peaks at 228.6 eV is the characteristic Mo^{4+} oxidation states of MoS_2, indicating more quasi MoS2 structure in sSMO NRs. Meanwhile, the peak located at 266.6 eV was more noticeable, which is corresponds to S 2s of MoS_2. For S 2p. the two main peaks located at 161.8 eV and 163.3 eV correspond to the S 2p3/2 and S 2p1/2 orbitals of MoS_2. Compared with mSMO NRs, more Mo^{4+} states of MoS_2 were formed in sSMO NRs with an increment increase of sulphur doping, resulting in the greater lattice stress.

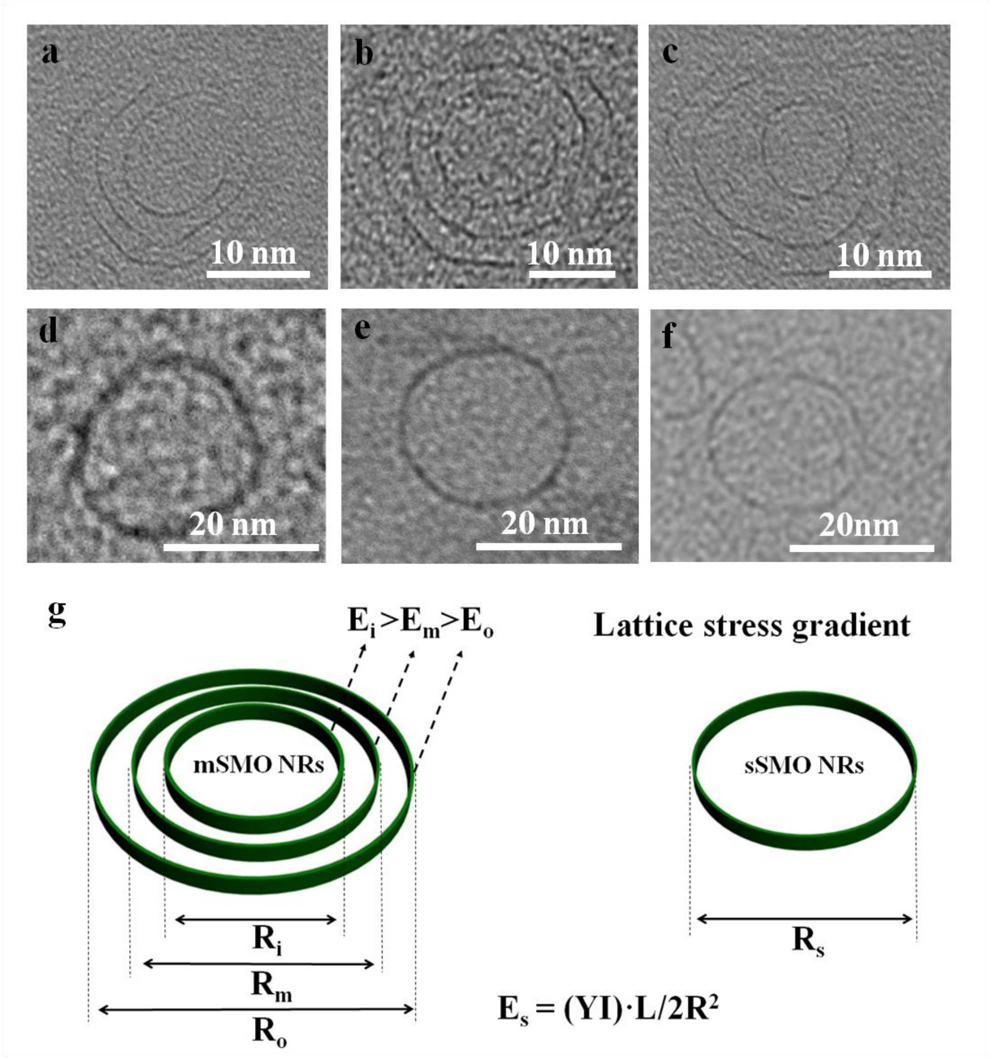

Supplementary Figure 12. (**a-c**) TEM images of the triple-level NRs; **(d-f)** TEM images of the single-level NRs; (**g**) Lattice stress gradient analysis for triple-level SMO NRs. Noted that a larger lattice stress will result in NRs with a smaller radius. For multi-level NRs, the lattice stress of NRs decreases gradually from the inside to the outside: $E_i > E_m > E_o$.

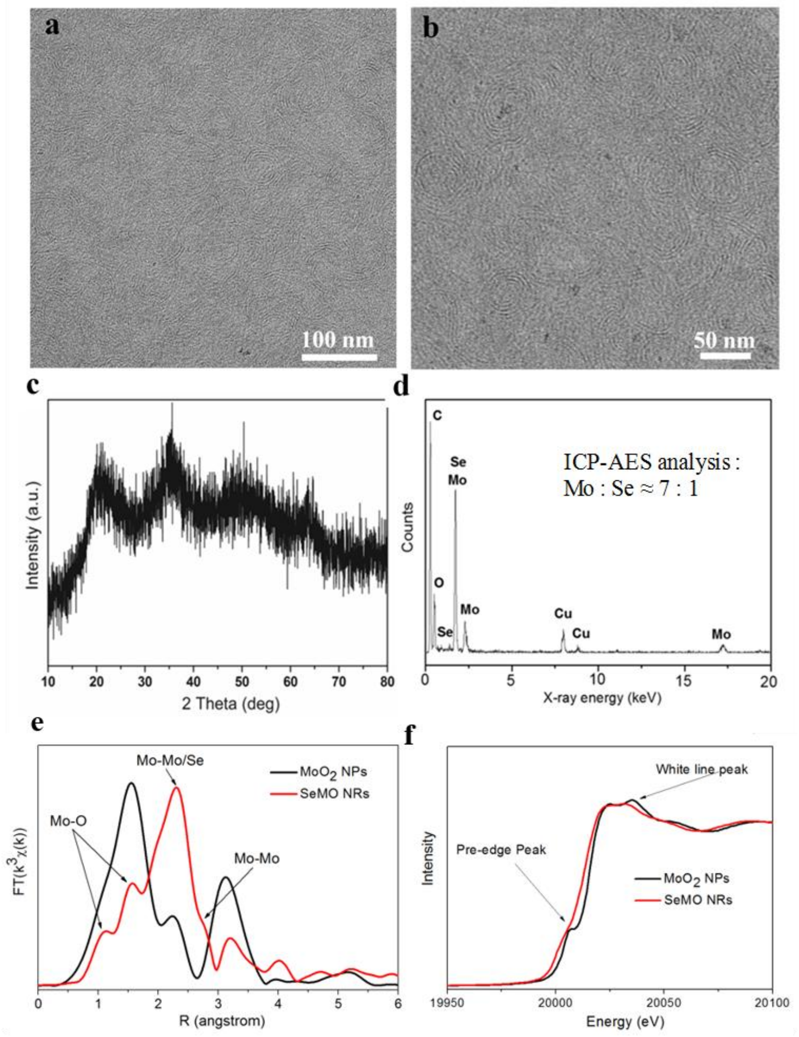

Supplementary Figure 13. (**a-b**) TEM images of the SeMo NRs; (**c-d**) XRD pattern and Energy-dispersive X-ray spectrums of SeMo NRs; (**e-f**) Fourier transforms of the Mo K-edge EXAFS patterns and XANES patterns of MoO_2 NPs and SeMO NRs.

Considering the larger atomic radius of Se, Se doping can generate bigger lattice stress, thus affecting their self-cyclization process. As a consequence, the product consists of NRs and nanorods and NRs structure with a shape selectivity is above 50 %. The XRD parttern with poor crystallinity is similar to sulfur doping, indicating the amorphous structure of the sample. The atomic ratio of Mo: Se is about 7: 1 based on the ICP-AES analysis. The preedge peak and white line peak observed in the XANES spectra show that the valence states of elements Mo is more close to $MoO_{2.}$ The first peak at around 1.0 Å and 1.5 Å corresponds to the Mo-O interaction in the first coordination structure, and the second peak at around 2.5 Å is due to the Mo-Mo/Se interaction in the second coordination structure. The peak at around 2.85 Å represents the Mo-Mo interaction in the second coordination structure (Supplementary Table 3).

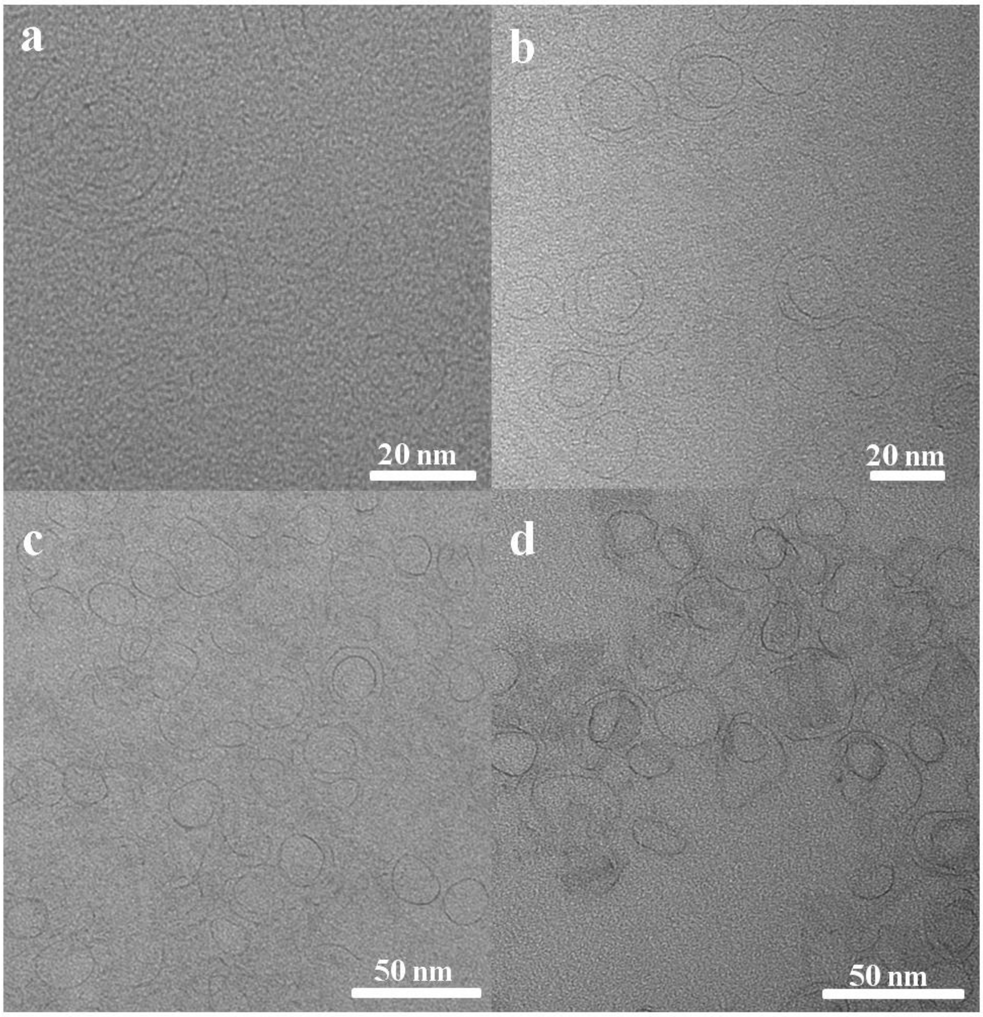

Supplementary Figure 14. TEM images of the mSMO NRs obtained at different growth durations. (**a**) 2 h, (**b**) 3 h, (**c**) 4 h and (**d**) 5 h.

In this synthetic system, there was no product at the initial stage of the reaction. Further prolonging the time to 2 hour, multi-level NRs structure could be formed. With increasing reaction time, the morphology of sample remains constant and the yields increase over time. This result shows that the self-cyclization and self-assembly spontaneously happen simultaneous during the synthetic solution.

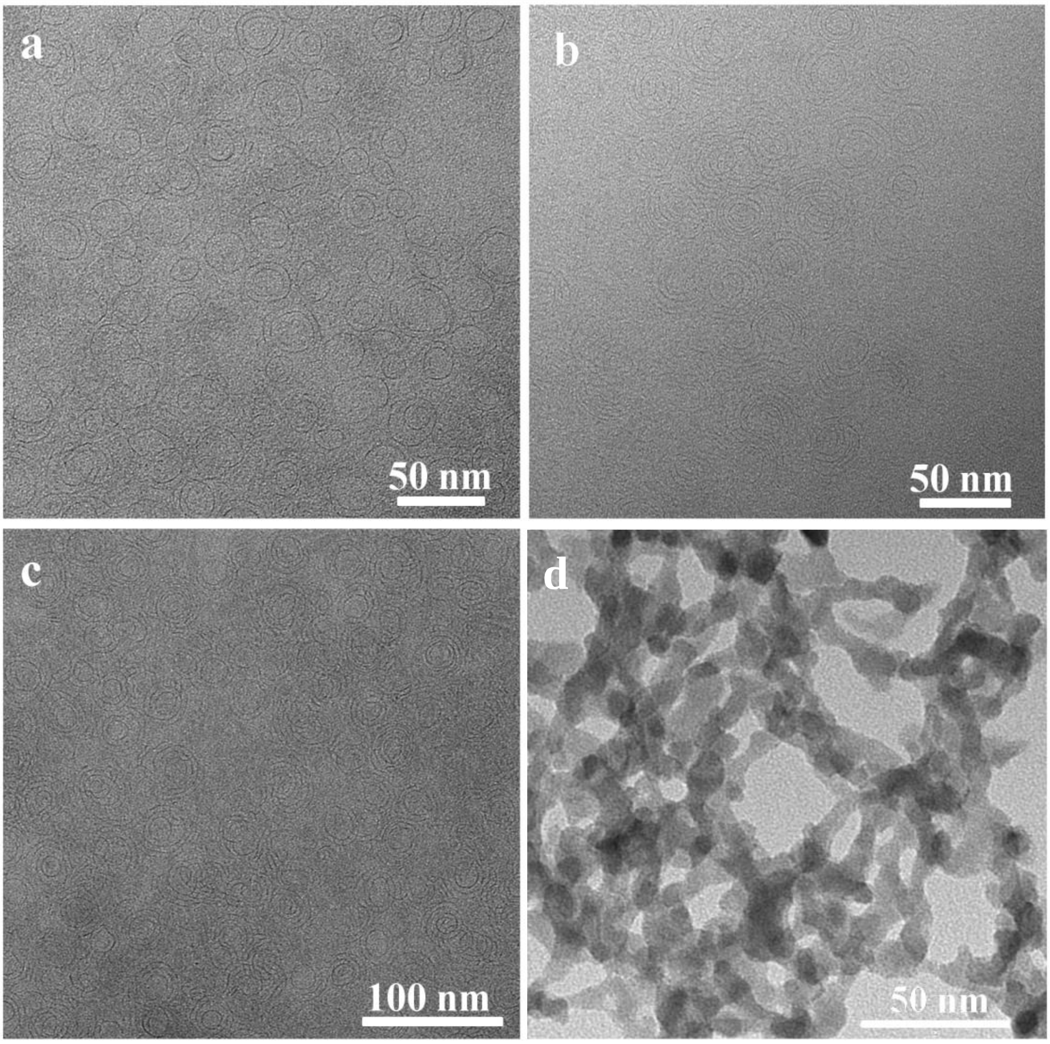

Supplementary Figure 15. (a-c)TEM images of the products obtained by using different alcohols; (**a**) 2-hexanol; (**b**) Octanol; (**c**) Dodecyl alcohol; (d) TEM image the samples without adding oleylamine.

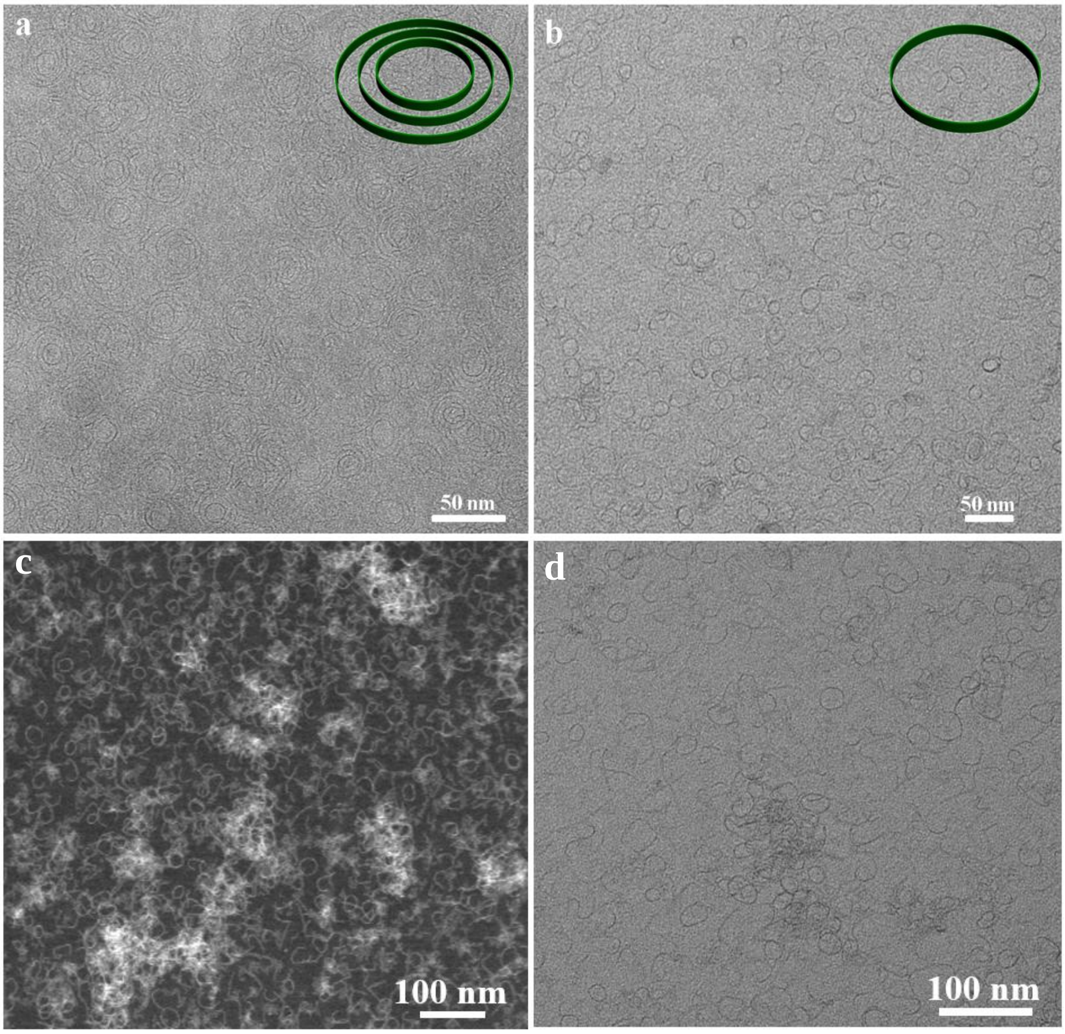

Supplementary Figure 16. (**a-b**) TEM images of the mSMO NRs before and after centrifugation treatment; (**c-d**) STEM and TEM images of the sample synthesized with high oleylamine ratio.

Based on the observation of TEM images before and after centrifugation, single-loop structure can be found, indicating that the non-bonding force between the NRs is very fragile. This ligand interactions on the surface of NRs could be destroyed after centrifugation, leading to the detachment of NRs.

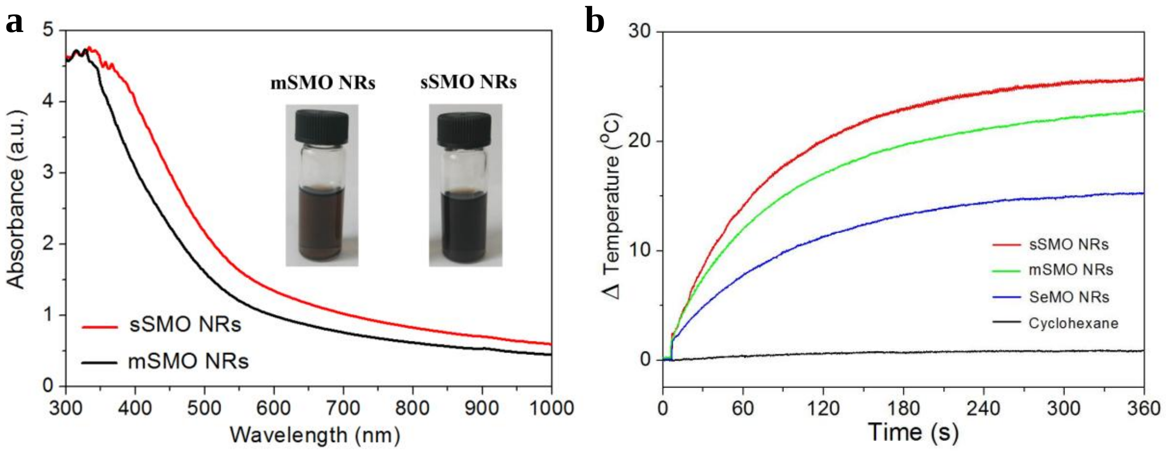

Supplementary Figure 17. (**a**) UV-Vis-NIR spectra of mSMO NRs and sSMO NRs dispersed in cyclohexane under the same concentration. Inset is the photographs of mSMO NRs and sSMO NRs dispersion; (**b**) Temperature recoreds of the mSMO NRs, sSMO NRs and SeMO NRs dispersion upon laser irradiation (1.0 W/cm^{-2}, 808 nm). The concentration of samlpes is 0.1 mg/mL^{-1}.

UV-Vis-NIR absorption spectra of sSMO NRs and mSMO NRs dispersion shows a broad absorption band spanning the visible and NIR regions. Compared with mSMO NRs, the UV-Vis-NIR absorption spectra of sSMO NRs displays enhanced optical absorption, indicating stronger light-absorption ability of sSMO NRs. It shall be noted that the sSMO NRs dispersion is dark yellow while the mSMO NRs dispersion is dark black, showing the different optical properties. The strong NIR-light absorption ability motivated us to study their photothermal effects. The temperature elevation shows the photothermal effect of the sample dispersed in cyclohexane under continuous laser irradiation. With the laser irradiation for 6 min, the temperature of pure cyclohexane almost not changed. Meanwhile, the temperature of sSMO NRs dispersion is higher than that of mSMO NRs and SeMO NRs. The temperature of the dispersions with sSMO NRs could be elevated by 26 °C in 6 minutes, indicating the sSMO NRs are remarkable photothermal transduction materials. The strong optical absorption allowed sSMO NRs to quickly and efficiently convert infrared laser energy into heat.

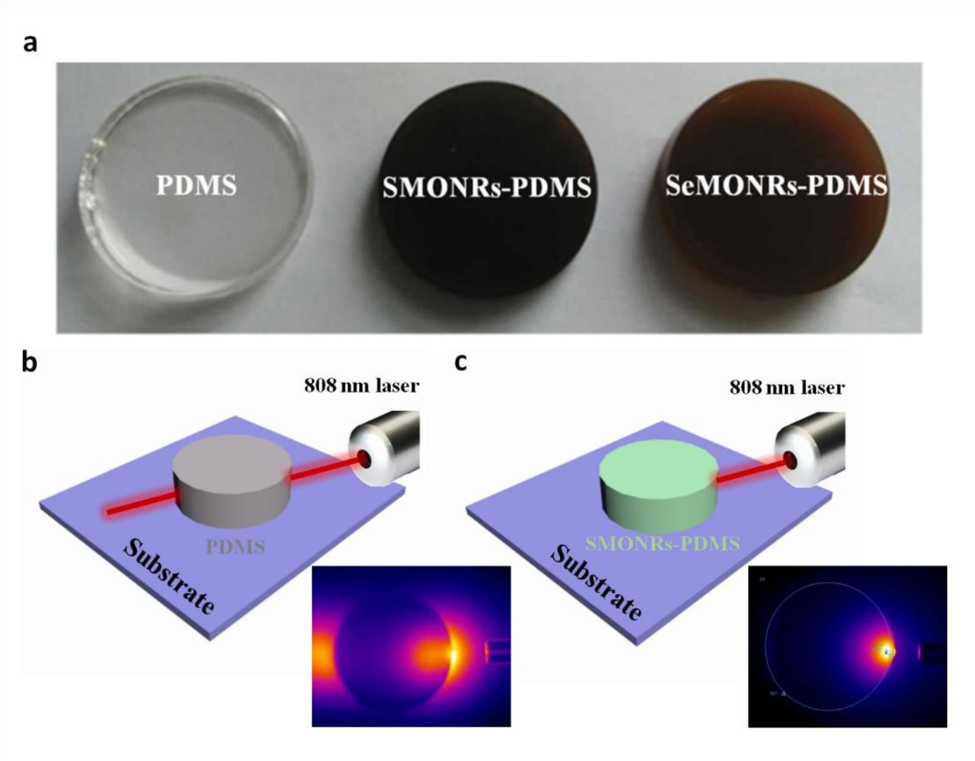

Supplementary Figure 18. (a) The photographs of pure PDMS, sSMONRs-PDMS and SeMONRs-PDMS composite; **(b-c)** Schematic diagram of pure PDMS and sSMONRs-PDMS composite irradiated by infaraed laser. The inset is monitored photothermal image of composite upon infrared laser irradiation.

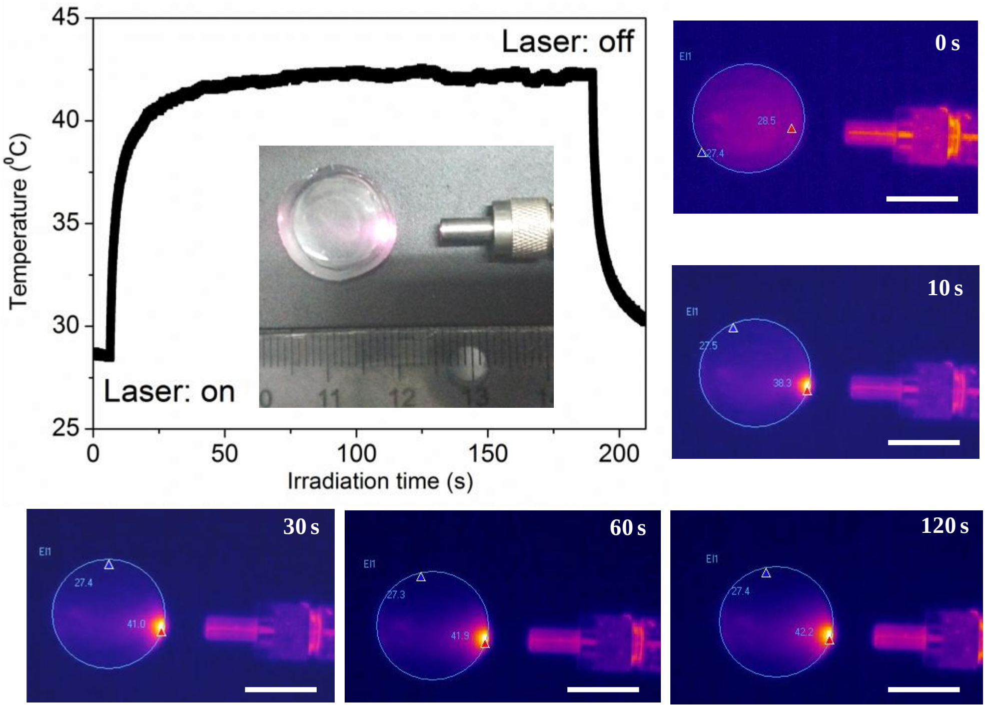

Supplementary Figure 19. Temperature recorded and photothermal images of different stages of pure PDMS under irradiation by laser under 808 nm laser with power density of 1.0 W/cm^{-2}. The inset is a digital photo of PDMS upon laser irradiation, indicating the optically transparent property. All scale bar: 1 cm.

Upon infrared laser irradiation, the temperature of PDMS increases from 27 °C to 42 °C at the beginning of the irradiation stage. Further prolonged irradiation time, the temperature is almost constant. It shall be noted that all recorded temperature value is a local maximum temperature. Based on the photothermal images of different stages, we found that the maximum temperature located the point of incident light. And the temperature of the whole material has not changed at all. We proposed that the rise of temperature could be attributed to the heat accumulation induced by infrared laser light. As heat quantity produced by light energy and thermal diffusion reach relative equilibrium state, the temperature will remain stable. As the laser is switched off, the temperature decreases rapidly.

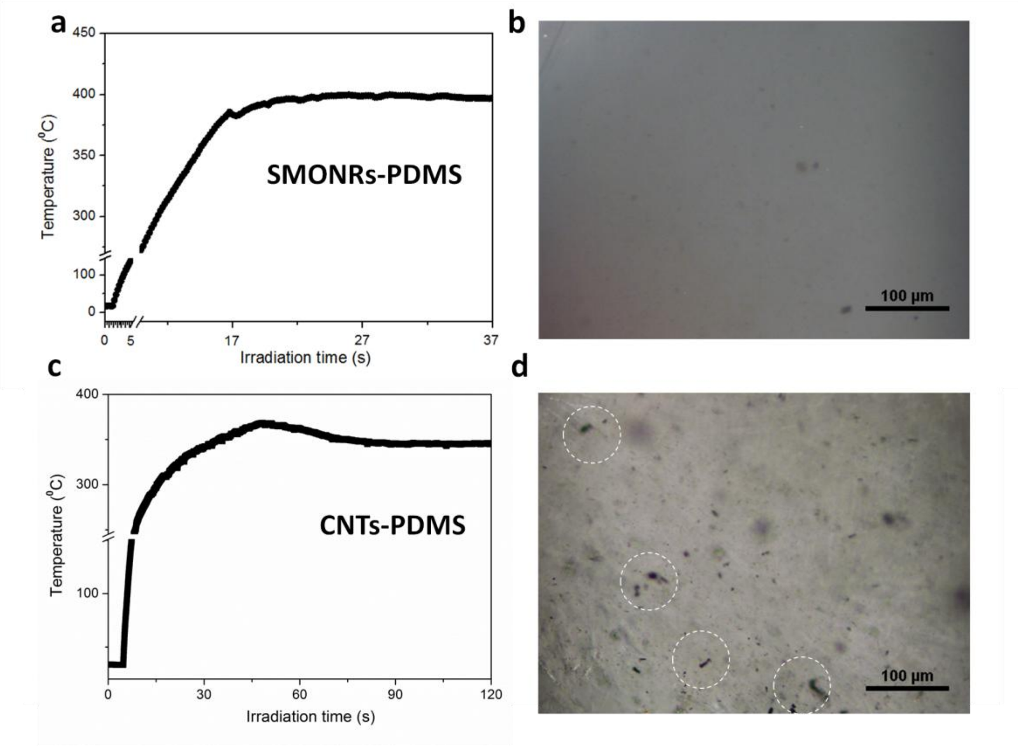

Supplementary Figure 20. (a) The monitored temperature profiles of sSMONRs-PDMS composite upon NIR laser irradiation (1.0 W/cm^{-2}, 808 nm); **(b)** Optical microscope photograph of sSMONRs-PDMS composite; **(c)** The monitored temperature profiles of CNTs-PDMS composite upon NIR irradiation; **(d)** Optical microscope photograph for CNTs-PDMS composite.

In order to compare the photothermal effect of carbon-based materials, we introduced commercial carbon nanotubes (CNTs) into PDMS polymer. As shown in optical microscope photograph, sSMO NRs show excellent dispersion with great compatibility. On the contrary, CNTs exhibit obvious aggregation in polymer. Obvious agglomeration of black blocks can be seen in CNTs-PDMS composite. Compared with sSMONRs-PDMS composite, CNTs-PDMS composite show lower temperature and relatively slower heating rate. The temperature of CNTs-PDMS composite heat up less than 400 in a minute under the same irradiation condition. Serious aggregation and poor dispersion of CNTs in PDMS impairs photothermal conversion and heat accumulation induced by NIR irradiation, thus affecting the photothermal effects.

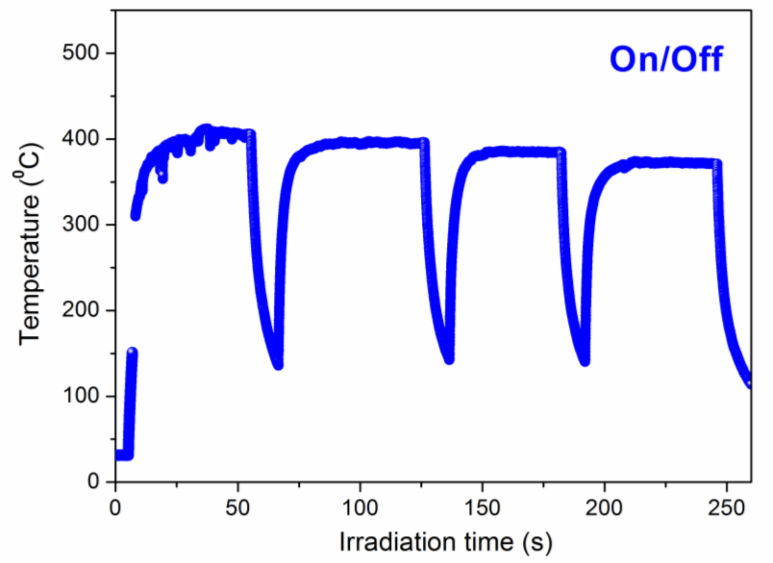

Supplementary Figure 21. Temperature records of sSMONRs-PDMS composite by switching on and off the laser irradiation (1.0 W/cm^{-2}, 808 nm). Noted that the break in the curve is ascribed to the transformation of temperature range for thermocouple thermometer. It can be seen that the temperature of sSMONRs-PDMS composite decrease slightly after switching on and off the laser irradiation, which could be ascribed to the structural damage and aggregation of NRs under high temperature.

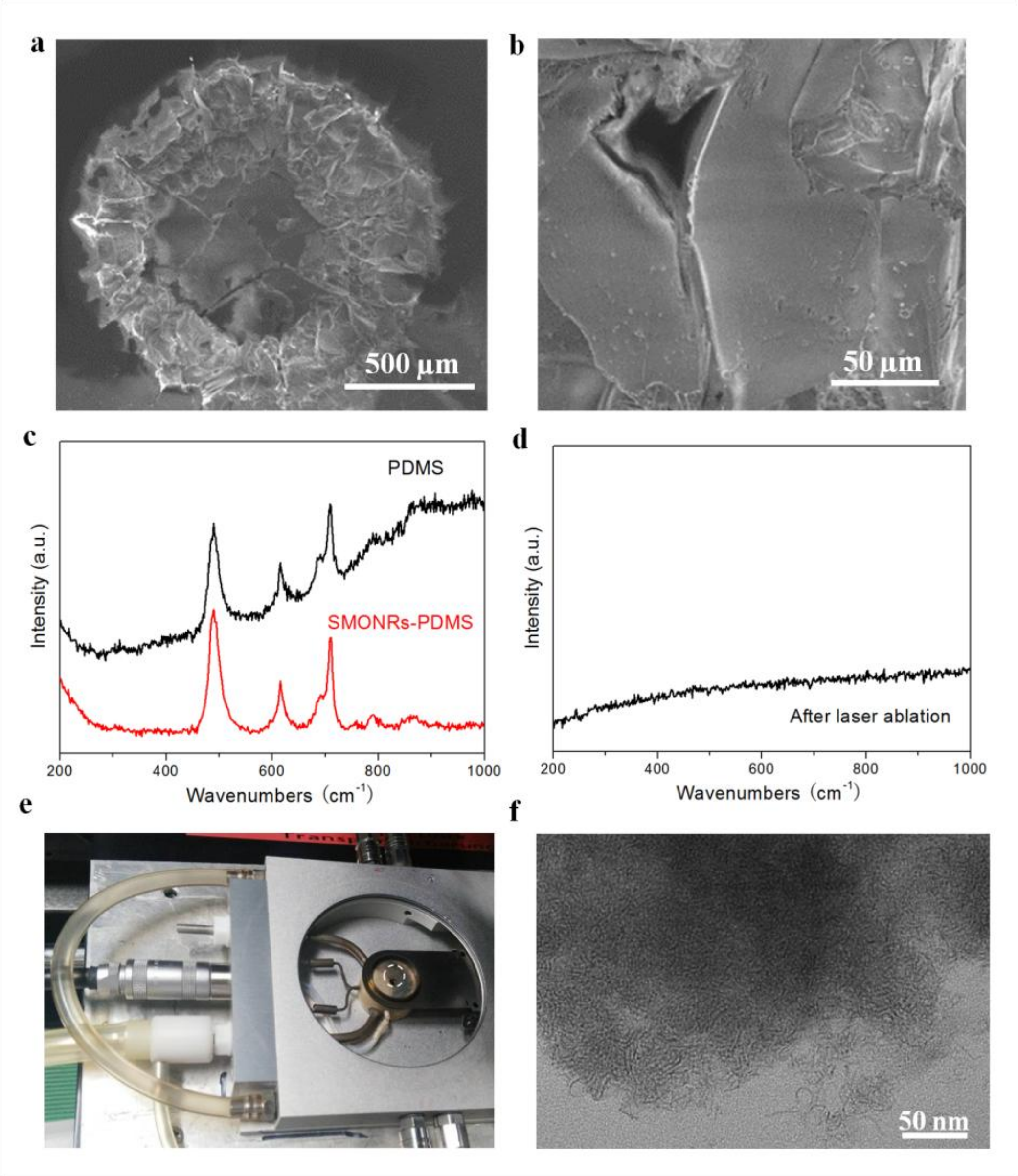

Supplementary Figure 22. (a-b) SEM images of composite after laser ablation; **(c-d)** Raman spectrum of sSMONRs-PDMS composite before and after laser ablation (633 nm); **(e)** Digital photo of heating platform with a heating rate 80 °C/min; **(f)** TEM image of sSMO NRs after rapid heating treatment.

The extremely high temperature lead to a centimeter-scale cavity after serious ablation phenomenon. Owing to the sSMO NRs embedded in PDMS polymer, it is difficult to acquire the morphology of NRs by TEM and SEM characterization after the smoke phenomenon. Raman spectrum was carried out to investigate the structural information of composite. According to the Raman spectrum of composite before and after irradiation, there was no Raman peaks after laser ablation, indicating that the polymer composite may be destroyed under high temperature. In addition,

we also conducted a rapid heating experiment to investigate the morphology of NRs. The resulting sSMO NRs was heated to 400 °C with a heating rate 80 °C/min on a heating platform. After heat treatment, some NRs structure had fractured into small pieces. Meanwhile, the decomposition of surface ligands results in a serious aggregation problem of NRs. Based on the discussion mentioned above, the extremely high temperature may result in the structural damage and aggregation of NRs in polymer after serious ablation phenomenon.

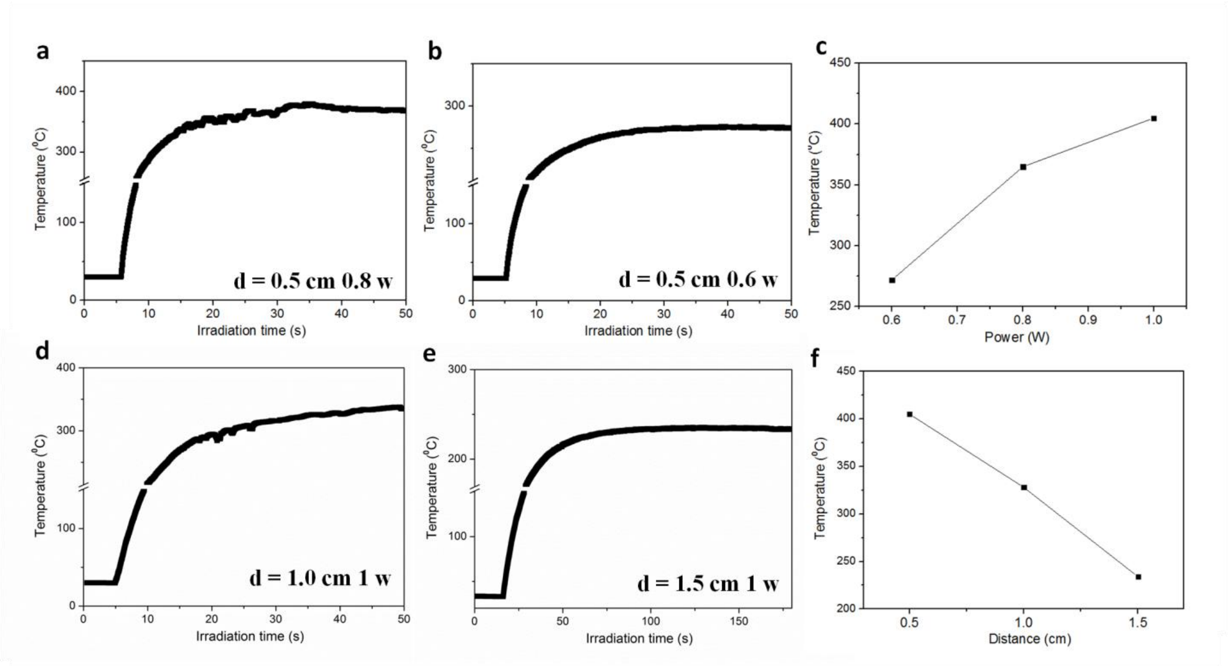

Supplementary Figure 23. (a-b) The monitored temperature profiles of sSMONRs-PDMS composite upon infrared laser irradiation under certain laser power density from left to right: 0.8 W/cm^{-2}, 0.6 W/cm^{-2}**; (c)** The relationship between temperature and laser power density for sSMONRs-PDMS composite; **(d-e)** The monitored temperature profiles of sSMONRs-PDMS composite with different irradiation distances. An 808 nm laser was used with power density of 1 W/cm^{-2}; (**f**) The relationship between temperature and irradiation distance for sSMONRs-PDMS composite.

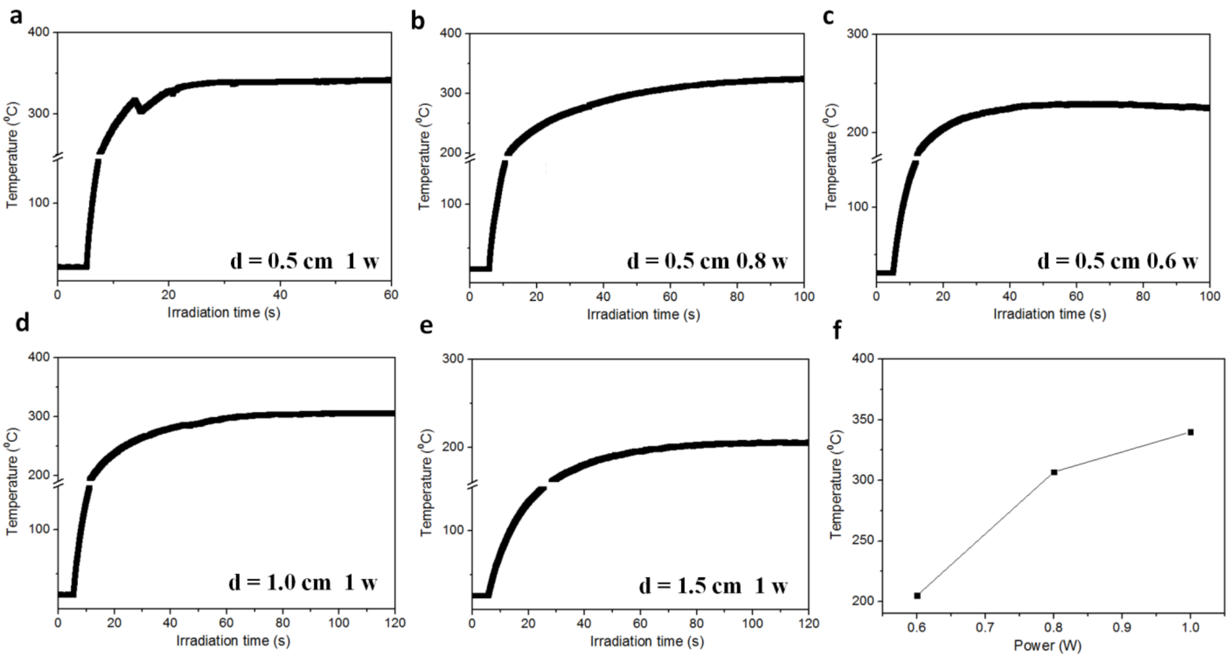

Supplementary Figure 24. (a-c) The monitored temperature profiles of mSMONRs-PDMS composite upon infrared laser irradiation under certain laser power density from left to right: 1 W/cm^{-2}, 0.8 W/cm^{-2}, 0.6 W/cm^{-2}**; (d-e)** The monitored temperature profiles of mSMONRs-PDMS composite upon laser irradiation with different irradiation distances between laser probe and composite; An 808 nm laser was used with power density of 1 W/cm^{-2}; **(f)** The relationship of temperature and laser power density for mSMONRs-PDMS composite.

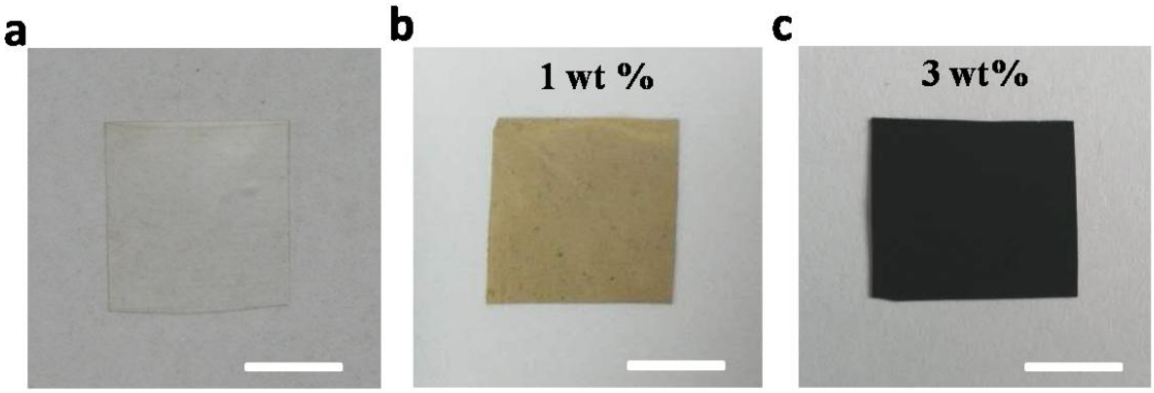

Supplementary Figure 25. (**a-c**) Photo images of pure vitrimer and sSMONRs-vitrimer with different concentrations. The scale bar is 1 cm. An increasing concentration of NRs deepens the color of composite.

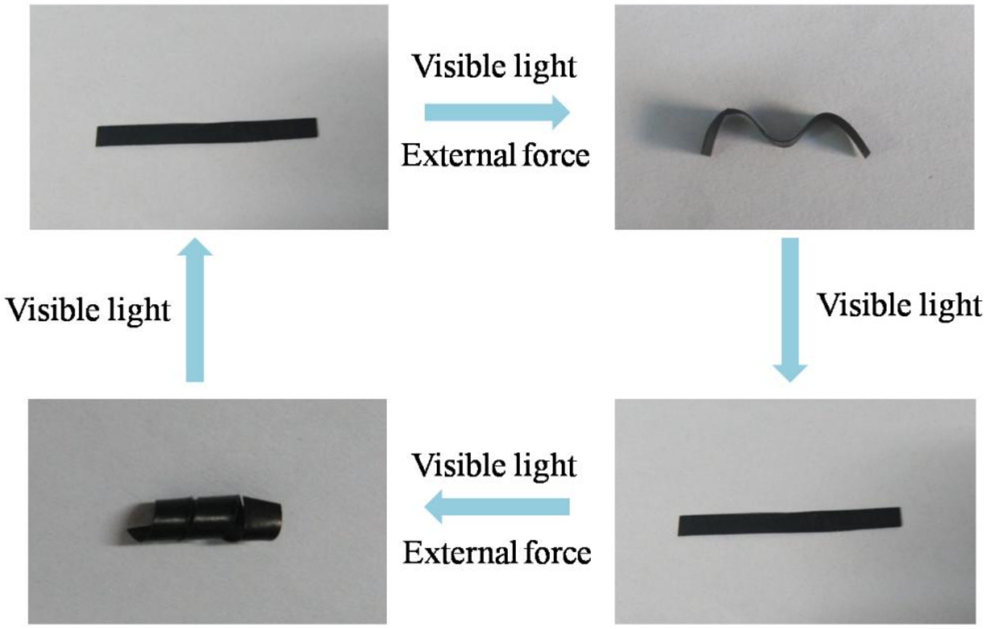

Supplementary Figure 26. Typical images of sSMONRs-vitrimer composite upon visible-light irradiation with an intensity of 140 mW/cm^2.

When a strip sample was exposed to visible light (1.47 W/cm^2) for 10 seconds, which induces the temperarure of the sample to ~70 °C (above T_g). The strip become soft and can be easily bent or folded into various structure. For example, a sSMONRs-vitrimer strip was bent into wave shape with the external force under irriration. After the light was turned off, wave-shaped structure process can be temporary retained. When the visible light was turned on, this wave-shaped structure can be quickly restored to their original strip shape. Overlaped structure can also return to initial structure in a few seconds, indicating the excellent shape memory feature of the sSMONRs-vitrimer under the visible light irradiation. This process can be well reshaped or reprocessed many times without any weakening.

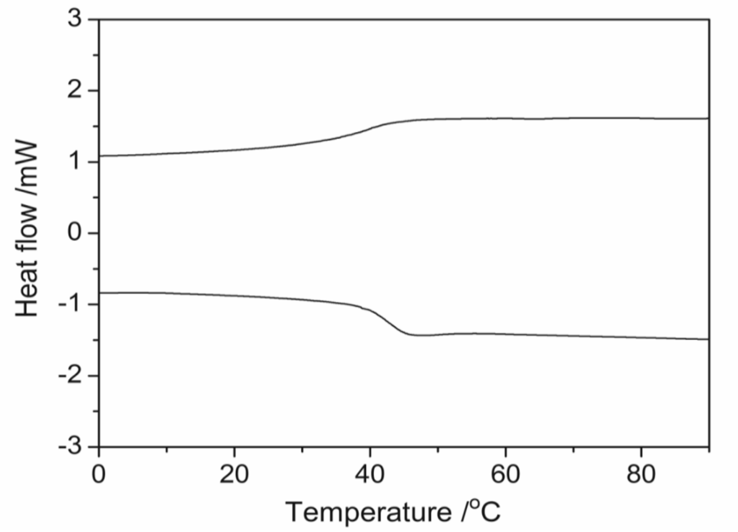

Supplementary Figure 27. DSC test of both heating/cooling of the sSMONRs-vitrimer (3 wt%), showing the the glass transition (T_g) of the sSMONRs-vitrimer is about 39 °C. The heating and cooling rate is 10 °C/min.

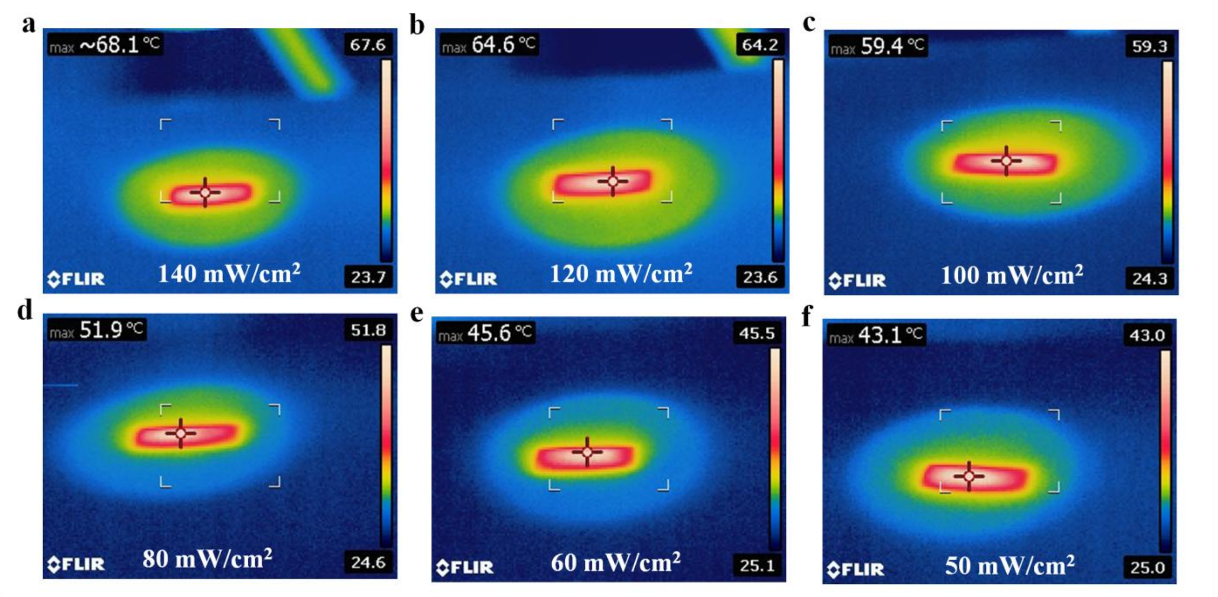

Supplementary Figure 28. (**a-f**) Photothermal images of the sSMONRs-vitrimer composite upon visible-light irradiation with certain laser power density. Photothermal images were captured under continuous irradiation and the temperature of the composite stay stable.

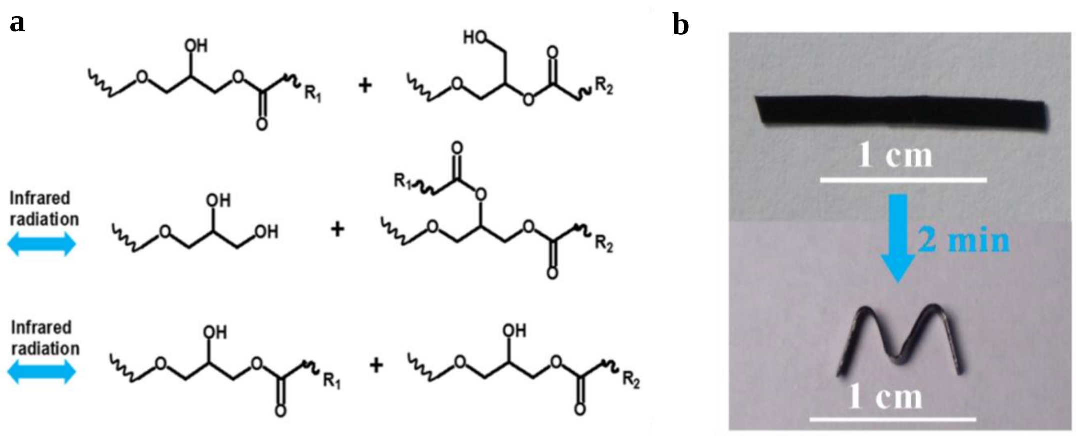

Supplementary Figure 29. (**a**) Illustration of light-induced transesterification reactions for SMONRs-vitrimer composite; (**b**) Reshaping of the sSMONRs-vitrimer composite upon infrared laser irradiation.

Upon infrared laser irradiation, as the temperature of sSMONRs-vitrimer is heated to above T_v, the transesterification will be activated. Under this conditions, an ester group and a hydroxyl group react with each other, producing a new ester and a new hydroxyl group which continue to react with other hydroxyl or ester groups to produce hydroxyl. Such a high temperature caused by infrared laser irradiation could be favorable for self-healing and reshaping applications. For example, a sSMONRs-vitrimer film was irradiated at the ridge under an intensity of 1.47 W/cm^2 for 2 min while the straight strip was bent into a “M” shape through the external force. As external stress was removed, it was found that the “M” shape did not change. The pattern can be well maintained even upon annealing at 250 °C, indicating that the new shape can be permanently reserved.

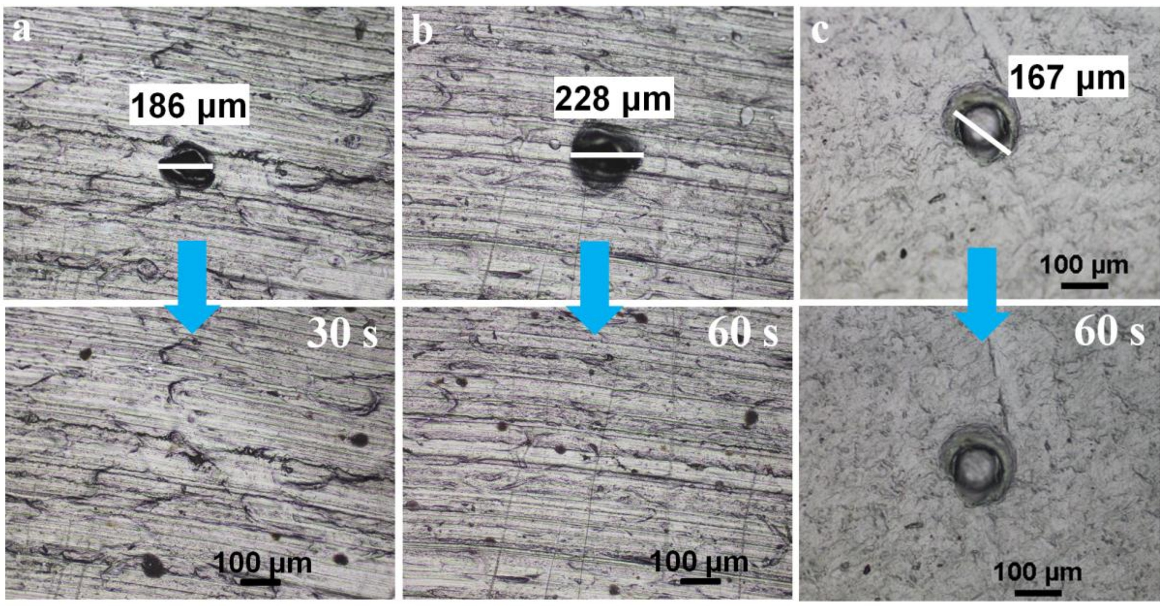

Supplementary Figure 30. Healing test of the sSMONRs-vitrimer composite by IR irradiation. (**a**) A needle pierced hole about 186 μm was healed by irradiation for 30 seconds (left); (**b**) A needle pierced hole about 228 μm was healed by irradiation for 60 seconds (middle), indicating that the healing time have close relationship of the size of hole; (**c**) Healing test of pure vitrimer by IR irradiation (right).

A typical hole 186 μm wide can be self-healed in situ in 30 s (808 nm, Intensity1.47 W/cm^{-2}), whereas a wider crack of 228 μm could still be healed with slightly longer irradiation times by IR irradiation. Therefore, the self-healing time is related to the size of the microcracks. By comparison, the pure vitrimer could not be healed for 1 min under the same intensity of light. Even for longer irradiation time, the pure vitrimer still could not be self-healed because the pure vitrimer sample can not absorb light.

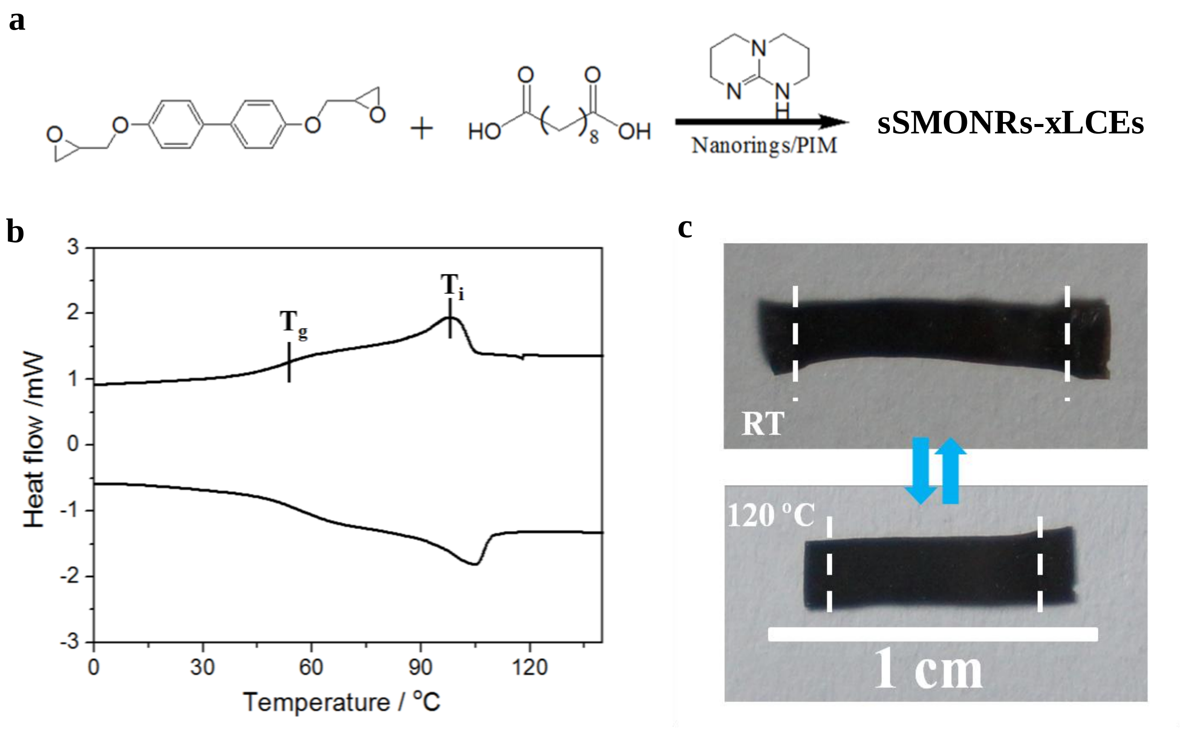

Supplementary Figure 31. (a) Synthesis of sSMONRs-xLCEs composite; (b) DSC test of both heating/cooling (rate of 10 °C/min) of sSMONRs-xLCEs composite; (c) Actuation of an aligned sSMONRs-xLCEs stripe.

Based on DSC test result, the glass transition (T_g) and the isotropic transition (T_i) of the sSMONRs-xLCEs were about 52 °C and 102 °C, respectively. The aligned sSMONRs-xLCEs strip can be stretched at high temperature above T_g and then cooled down to room temperature. This aligned SMONRs-xLCEs film can reversibly stretch and contract between the isotropic phase and the liquid crystal phase. The elongation percentage can be calculated about 20 %.

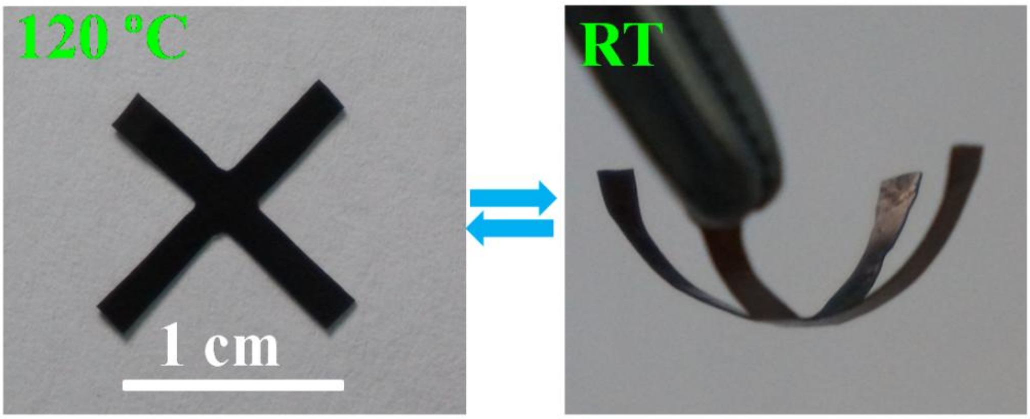

Supplementary Figure 32. Examples of dynamic 3D structures of the sSMONRs-xLCEs composite.

The sample was prepared by streching and irridiating each petal one by one. Samples were fristly processed into a four-petal film. Each petal could be processed into bending mode one by one. Reversible bending movement can be achieved by controlling the amount of light energy to the film, leading to a temperature gradient along the thickness the film. Therefore, there is a reduction on the extent of alignment. Once external light is removed, the film will bend toward the bottom part becaused of the large actuation amplitudes.

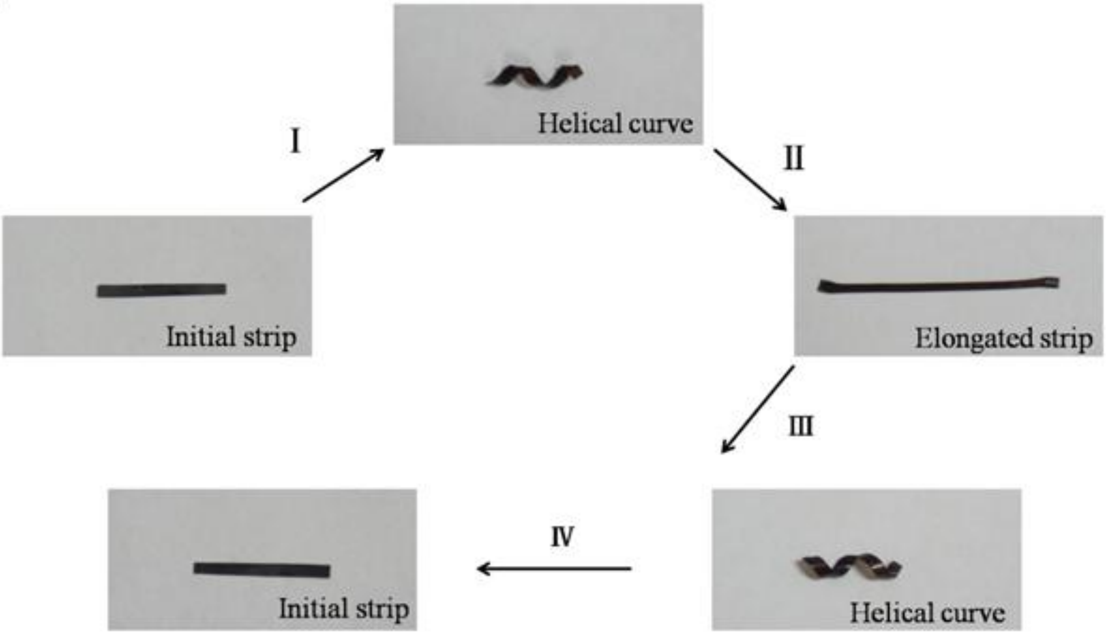

Supplementary Figure 33. Triple shape memory of the sSMONRs-xLCEs composite.

Triple shape memory of the sSMONRs-xLCEs composite can be well demonstrated from initial strip, to helical curve, to elongated strip, to recover their initial strip through sequential heating treatment; Ⅰ: A flat initial strip was deformed into helical curve, and the helical shape was fixed after cooling down to 75 ºC; Ⅱ: Helical curve was further deformed into elongated strip through the external force at this temperature, then cool to room termperature; Ⅲ: By heating, the temperature of sSMONRs-xLCEs composite is above T_g, recovering to helical curve; Ⅳ: Further increasing the temperature above $T_{i,}$ then the sSMONRs-xLCEs composite restore to initial strip.

Supplementary Table 1. The atomic ratio of samples determined by ICP-OES.

The atomic ratio	mSMO NRs	sSMO NRs	SeMO NRs
Mo : S	5 : 1	2.2 : 1	
Mo : Se			7 : 1

Supplementary Table 2. Refined EXAFS structural parameters of Mo atoms; Type of bond, Bond distance R, Coordination number N, and Debye–Waller factor, σ^2.

Sample	Bond	Bond length	N	$\sigma^2 (10^{-3}\ Å^2)$
MoO_2 NPs	Mo-O	1.68	1.5	4.6
	Mo-O	2.0	2.4	3.9
	Mo-Mo	2.57	0.7	6.6
mSMO NRs	Mo-O	1.59	1.1	4.1
	Mo-O	2.01	2.0	3.2
	Mo-S	2.41	1.0	11.9
	Mo-Mo	2.60	0.8	4.7
	Mo-Mo	3.01	1.0	10.9
Reference compound MoO_2 foil	Mo-O	1.75	2	3.6
	Mo-O	2.03	4	1.1
	Mo-Mo	2.55	1	3.7
Reference compound MoO_3 foil	Mo-O	1.63	2	7.4
	Mo-O	1.82	2	1.7
	Mo-O	2.23	2	1.0

Supplementary Table 3. Refined EXAFS structural parameters of Mo atoms; Type of bond, Bond distance R, Coordination number N, and Debye–Waller factor, σ^2.

Sample	Bond	Bond length	N	$\sigma^2(10^{-3}$ Å$^2)$
MoO_2 NPs	Mo-O	1.68	1.5	4.6
	Mo-O	2.0	2.4	3.9
	Mo-Mo	2.57	0.7	6.6
SeMO NRs	Mo-O	1.61	0.6	4.7
	Mo-O	2.02	1.9	5.6
	Mo-Se	2.50	0.9	12.5
	Mo-Mo	2.53	1.2	9.2
	Mo-Mo	3.02	1.1	10.3
Reference compound MoO_2 foil	Mo-O	1.75	2	3.6
	Mo-O	2.03	4	1.1
	Mo-Mo	2.55	1	3.7
Reference compound MoO_3 foil	Mo-O	1.63	2	7.4
	Mo-O	1.82	2	1.7
	Mo-O	2.23	2	1.0
