# Supplementary material for: Atomic-level molybdenum oxide nanorings with full-spectrum absorption and photoresponsive properties
Source: Nat Commun. 2017 Nov 16;8:1559. doi: 10.1038/s41467-017-00850-8 (PMC5691127; doi:10.1038/s41467-017-00850-8)
Supplement: Supplementary file 2 — Description of Additional Supplementary Files [file 41467_2017_850_MOESM2_ESM.pdf]

Description of Additional Supplementary Files

File Name: Supplementary Movie 1
Description: Irradiation of PDMS-nanoring composites by NIR laser.

File Name: Supplementary Movie 2
Description: Shape memory effects of the composites irradiated by visible light.
